# Insights into the Molecular Mechanisms of Alzheimer’s and Parkinson’s Diseases with Molecular Simulations: Understanding the Roles of Artificial and Pathological Missense Mutations in Intrinsically Disordered Proteins Related to Pathology

**DOI:** 10.3390/ijms19020336

**Published:** 2018-01-24

**Authors:** Orkid Coskuner-Weber, Vladimir N. Uversky

**Affiliations:** 1 Türkisch-Deutsche Universität, Theoretical and Computational Biophysics Group, Molecular Biotechnology, Sahinkaya Caddesi, No. 86, Beykoz, Istanbul 34820, Turkey; 2 Department of Molecular Medicine and USF Health Byrd Alzheimer’s Research Institute, Morsani College of Medicine, University of South Florida, Tampa, FL 33612, USA; 3 Laboratory of New Methods in Biology, Institute for Biological Instrumentation, Russian Academy of Sciences, 142290 Pushchino, Moscow Region, Russia

**Keywords:** genetics, artificial mutation, intrinsically disordered protein, Alzheimer’s disease, Parkinson’s disease, molecular dynamics simulations

## Abstract

Amyloid-β and α-synuclein are intrinsically disordered proteins (IDPs), which are at the center of Alzheimer’s and Parkinson’s disease pathologies, respectively. These IDPs are extremely flexible and do not adopt stable structures. Furthermore, both amyloid-β and α-synuclein can form toxic oligomers, amyloid fibrils and other type of aggregates in Alzheimer’s and Parkinson’s diseases. Experimentalists face challenges in investigating the structures and thermodynamic properties of these IDPs in their monomeric and oligomeric forms due to the rapid conformational changes, fast aggregation processes and strong solvent effects. Classical molecular dynamics simulations complement experiments and provide structural information at the atomic level with dynamics without facing the same experimental limitations. Artificial missense mutations are employed experimentally and computationally for providing insights into the structure-function relationships of amyloid-β and α-synuclein in relation to the pathologies of Alzheimer’s and Parkinson’s diseases. Furthermore, there are several natural genetic variations that play a role in the pathogenesis of familial cases of Alzheimer’s and Parkinson’s diseases, which are related to specific genetic defects inherited in dominant or recessive patterns. The present review summarizes the current understanding of monomeric and oligomeric forms of amyloid-β and α-synuclein, as well as the impacts of artificial and pathological missense mutations on the structural ensembles of these IDPs using molecular dynamics simulations. We also emphasize the recent investigations on residual secondary structure formation in dynamic conformational ensembles of amyloid-β and α-synuclein, such as β-structure linked to the oligomerization and fibrillation mechanisms related to the pathologies of Alzheimer’s and Parkinson’s diseases. This information represents an important foundation for the successful and efficient drug design studies.

## 1. Introduction

### 1.1. A Brief Introduction to Parkinson’s Disease and Pathological Roles of α-Synuclein

Parkinson’s disease (PD) is the most common age-related movement disorder and the second most common neurodegenerative malady after Alzheimer’s disease (AD), affecting 1–2% of the population over the age of 65. It involves the loss of dopaminergic neurons in the *Substancia nigra*, eventually leading to the decreased dopamine levels in the striatum that causes characteristic PD symptoms, such as tremors, rigidity of muscles and bradykinesia. While the exact mechanism of PD pathogenesis is not completely understood, aggregation of the presynaptic protein α-synuclein (αS) is believed to play a crucial role in the etiology of this malady [1,2,3]. Being a typical intrinsically disordered protein, αS does not have a single biological function but possesses a multitude of functional activities [4,5,6,7,8]. For example, a significant fraction of αS is involved in interaction with membrane, especially synaptic vesicles associated with the vesicular transport processes. These observations suggest that αS plays a critical role in vesicular trafficking [9,10,11,12,13,14,15,16,17,18,19,20].

The wild-type (WT) αS is a 140 amino acid-long protein, which is intrinsically disordered under physiological conditions. There are no Trp residues in αS. Experimental studies have shown that monomeric αS has a more compact structure than expected for a completely unfolded protein and this compactness has been linked to inhibition of fibrillation due to burial of the NAC region [21,22,23,24,25]. Small angle X-ray scattering analysis showed that the radius of gyration (R_g_)—which is utilized to describe the dimensions of the protein chain—is about 40 Å, which is much larger than that for a folded globular protein of 140 residues but significantly smaller than that for a fully unfolded random coil polypeptide [26]. Nuclear magnetic resonance (NMR) studies showed that αS adopts an ensemble of conformations that are stabilized by long-range interactions [21]. Specifically, a long-range intramolecular interaction between the C-terminal region (residues 120–140) and the central part of αS (residues 30–100) was noted [21]. This interaction was proposed to inhibit fibrillation and could arise from electrostatic or hydrophobic or both types of interactions. The amino acid sequence of human αS is shown below:

MDVFMKGLSKAKEGVVAAAEKTKQGVAEAAGKTKEGVLYVGSKTKEGVVHGVATVAEKTKEQVTNVGGAVVTGVTAVAQKTVEGAGSIAAATGFVKKDQLGKNEEGAPQEGILEDMPVDPDNEAYEMPSEEGYQDYEPEA

Standard tools of structural biology have failed to provide the 3D structures of the monomers and the oligomers of αS and Aβ in aqueous solution at the atomic level. αS is an acidic intrinsically disordered protein (IDP) with three domains; namely N-terminal lipid-binding domain, amyloid-binding central domain (NAC) and C-terminal acidic tail [11,27,28,29,30,31,32,33]. αS can be present as an α-helical structure in association with phospholipids or an unfolded conformation in the cytosol, suggesting that it plays specific roles in different cellular locations based on its dynamic structure. The N-terminal domain of αS (residues 1–87) is a positively charged region that includes seven 11-amino acid repeats. Each of these repeats contains a highly conserved KTKEGV hexameric motif that is also present in the α-helical domain of apolipoproteins. Furthermore, the ability of αS to disrupt lipid bilayers is related to these repeat sequences. These repeats, via their ability to induce αS helical structure and subsequently reduce the tendency of αS to form β-structures, are important in αS and lipid interactions.

The core region of αS (residues 61–95), also known as NAC, is involved in fibril formation and aggregation as it can form cross β-structures. The C-terminal domain of αS (residues 96–140) is an acidic tail of 43-amino acid residues, containing 10 Glu and 5 Asp residues. This C-terminal region contains three of the four tyrosine residues. Structurally, the C-terminal domain of αS is present in a random coil structure due to its low hydrophobicity and high net negative charge. In vitro studies have revealed that αS aggregation can be induced by reduction of pH which neutralizes these negative charges. An interaction between the C-terminal domain and the NAC region of αS is thought to be responsible for inhibition of αS aggregation [11,27,28,29,30,31,32,33,34]. Furthermore, in the presence of Al^3+^, the C-terminal domain of αS binds to this metal ion and thus the ruined inhibitory effect of the C-terminal on NAC leads to αS aggregation [35]. The phosphorylation of serine 129 is important in the inhibitory property of the C-terminal region as dephosphorylation of serine 129 causes αS aggregation. The C-terminal of αS is homologous to the small heat shock proteins (HSPs), suggesting a protective role for αS in keeping proteins out of the aggregation and degradation processes. However, the 3D structure of monomeric and oligomeric αS in aqueous solution and how artificial as well as genetic mutations impact monomeric and oligomeric αS could not be investigated using experiments at the atomic level with dynamics. These genetic mutations may require further development in drug studies associated with “personalized medicine” (see above).

To investigate the mechanisms defining these long-range interactions, introduction of the artificial mutations to the αS sequence is needed. Artificial mutations can provide structural scaffolds to modulate the pathological/biological activities of a variety of target intrinsically disordered proteins, including αS. These mutations can also help in better understanding of the specifics of binding of a query protein to target molecules, such as small molecules/drugs.

### 1.2. α-Synuclein Mutations and Parkinson’s Disease

Besides artificial mutations that are introduced to understand structural features and functional mechanisms, there are several natural genetic variations that play a role in the pathogenesis of PD. Familial Parkinsonism accounts for a significant proportion of cases of PD and is related to specific genetic defects that are inherited in a dominant or recessive pattern [36,37]. The entire set of genes responsible for the familial Parkinsonism is not yet fully known, although several loci have been identified in the last years [37]. The clinical phenotype presented by patients with familial Parkinsonism is variable. Not infrequently, in addition to the typical symptoms of PD, many of these patients present other deficits, such as dystonia, dysautonomia, cognitive and behavioral changes, sleep disorders and perceptive deficits. Dementia is not uncommon in patients with PD, both sporadic and familial.

### 1.3. A53T Mutation

Although PD is non-hereditary in the majority of cases, several kindreds with hereditary forms have been long reported, particularly by Herman Lundborg in 1913 and by Henry Mjönes in 1949, in Sweden (see [38,39] and references therein). Golbe et al. described large Italian-American kindred with autosomal dominant parkinsonism originating from the town of Contursi (Southern Italy) [40] and Markopolou et al. showed a similar phenotype in the Greek-American family [41]. In 1997, the A53T mutation in the αS gene (*SNCA*) was found to be linked to PD in members of the Contursi family and in three families from Greece (see [42] and references therein). Subsequent work revealed that αS is a principal component of LB in brains from patients with αS A53T mutation [43], as well as in sporadic PD [44]. The A53T mutation of αS has since been detected in several additional Greek families and in patients of Greek origin residing in Australia and Germany [45,46,47,48,49]. Only few individuals without known Greek or Italian ancestry have so far been reported to carry αS A53T mutation. For instance, one patient from the United Kingdom [50], now deceased, displayed symptoms consistent with sporadic late-onset PD and two affected members of a Korean family were investigated and their haplotype was different from the Greek/Contursi haplotype [51]. Puschmann et al. reported in 2009 a family from southern Sweden with αS A53T mutation [52]. In vitro, αS protein with the A53T mutation are more prone to form fibrils than the WT αS protein [53,54,55]. The exact structural and functional mechanism of the effects of mutations on pathological transformation of αS at the atomic level could not be investigated using experiments.

### 1.4. A30P Mutation

Krüger et al. initiated a detailed mutation analysis of five translated *SNCA* exons in 192 sporadic PD cases and in 7 unrelated patients with a family history of PD [56]. They conducted single strand conformation polymorphism (SSCP) analysis of the coding exons 3 to 7 and reported an SSCP band shift analyzing exon 3 of a single patient with familial PD. This analysis identified a G→C transversion at nucleotide position 88 of the coding sequence, which causes A30P mutation in the αS protein. The mother of the patient presented with symptoms at age 56 and was diagnosed with PD according to the UK PD Society Brain Bank. More family members including a sibling and a child of the index patient were carriers of αS A30P mutation [56]. Again, the detailed structure and function relationship of αS A30P mutant could not be investigated at the atomic level with dynamics using experiments due to fast dynamical changes, rapid aggregation mechanism and solvent effects.

### 1.5. E46K Mutation

In PD, whether sporadic or familial, the most common form of dementia is the dementia with Lewy Bodies (DLB) characterized by the presence of Lewy bodies in neocortical and paralimbic areas and AD-type lesions. DLB is a heterogeneous disease with variable clinical and pathological features typically characterized by dementia, visual hallucinations, Parkinsonism and fluctuations in cognition and attention associated with the presence of Lewy bodies in a pattern more widespread than that usually observed in the brains of PD patients. The clinical diagnostic criteria of DLB proposed by an international consortium include the presence of cognitive decline plus spontaneous parkinsonian symptoms and signs, visual hallucinations and fluctuations in consciousness early in the course of the disease [37,42]. The cause of DLB is likely to be related to multiple factors. Most cases are sporadic but familial cases have been described as well. Genetic investigation of familial DLB has been very limited until Zarranz et al. reported a family from the Basque Country with autosomal dominant parkinsonism, possible clinical criteria and typical pathological features of DLB, which was produced by a novel E46K mutation of αS [57].

### 1.6. H50Q and G51D Mutations

Proukakis et al. amplified and sequenced *SNCA* exons in DNA extracted from *substancia nigra* of 5 Queen Square PD Brain Bank cases [58]. They detected a point mutation in exon 3 in 1 case, causing a nonconservative missense change of the histidine to the polar uncharged glutamine (H50Q) [58]. The patient, a Caucasian English female presented at age 71 tremor, responded to L-dopa, became forgetful at age 80 and died at age 83 [58]. Lesage et al. presented in 2013 detailed clinical, neuropathological and functional data concerning a French family with parkinsonian-pyramidal syndrome associated with a heterozygous G51D mutation in the αS protein [59]. Although these point mutations and genomic multiplications are rare, they led to the important discovery that αS is the major fibrillar component of LBs and Lewy neurites (LN), the pathological hallmarks of PD in both familial and sporadic cases.

### 1.7. Some Approaches to the PD Treatment

There are many medications available to treat the symptoms of PD, although none of the existing drugs can actually reverse the disease. The most potent medication for PD is levodopa [60,61]. Plain levodopa produces nausea and vomiting. A combination with carbidopa prevents these side effects. The well-known combined carbidopa/levodopa formulation is called Sinemet^®^ [62]. The addition of carbidopa prevents levodopa from being converted into dopamine in the bloodstream, allowing more of it to get into the brain. Usually, a small dose of levodopa is needed to treat symptoms. With increased dosing and prolonged use of levodopa, patients experience side effects, such as dyskinesia (spontaneous, involuntarily movement) and “on-off” periods when the medication suddenly and unpredictably starts or stops working.

Dopamine agonists are drugs that stimulate the parts of the human brain that are affected by dopamine. In fact, the brain is tricked into believing that it is receiving the dopamine it needs. Dopamine agonists are not as potent as carbidopa/levodopa and therefore less likely to cause dyskinesia. The two most common agonists in the US are pramipexole (Mirapex) and ropinirole (Requip) Neupro^®^, which was re-approved after several years of being off the market [63,64,65,66]. Parlodel^®^ is available but less commonly utilized. Dopamine agonists cause nausea, hallucinations, sedation and lightheadedness due to low blood pressure. Apokyn is a powerful medication that promptly relieves PD symptoms within minutes but only provides 30 to 60 min of benefit [67].

Anticholinergics can be helpful for tremor treatment and may ease dystonia associated with wearing-off or peak-dose effects [68]. They have little effect on other symptoms of Parkinson’s. Artane^®^ and Cogentin^®^ are examples of this class. These do not act directly on the dopaminergic system but these drugs decrease the activity of acetylcholine, a neurotransmitter that regulates movement. Potential adverse effects include blurred vision, dry mouth, constipation and urinary retention. MAO-B inhibitors block an enzyme in the brain that breaks down levodopa [69]. COMT inhibitors, such as Comtan^®^ and Tasmar^®^ represent a class of Parkinson’s medications [70]. These agents have no direct effect on PD symptoms but instead are used to prolong the effect of levodopa by blocking its metabolism. COMT inhibitors are used primarily to help with the problem of wearing-off, in which the effect of levodopa becomes short-lived. Furthermore, Symmetrel is a mild agent that is used in early PD to help ease tremors [71]. In recent years, amantadine has also been found useful in reducing dyskinesias that occur with dopamine medication [72]. Rivastigmine (Exelon) is the only medication approved by the US Food and Drug Administration for the treatment of dementia in PD [73].

Currently, there are two surgical treatments available for people living with PD—deep brain stimulation (DBS) and surgery performed to insert a tube in the small intestine, which delivers a gel formulation of carbidopa/levodopa (Duopa™) [74,75,76,77]. In DBS, surgery is performed to insert electrodes into a targeted area of the brain, using MRI and recordings of brain cell activity during the procedure. A second procedure is performed to place an implantable pulse generator or IPG (similar to a pacemaker) under the collarbone or in the abdomen. The IPG provides an electrical impulse to a part of the brain involved in motor function. Those who undergo DBS surgery are given a controller to turn the device on or off. DBS is certainly the most important therapeutic advancement since the development of levodopa. It is most effective for individuals who experience disabling tremors, wearing-off spells and medication-induced dyskinesias, with studies showing benefits lasting at least five years. That said, it is not a cure and it does not slow PD progression. Furthermore, DBS carries a small risk of infection, stroke, bleeding or seizures. DBS surgery may be associated with reduced clarity of speech. A small number of people with PD have experienced cognitive decline after DBS surgery.

Carbidopa/levodopa enteral suspension (Duopa™) is a gel formulation of the gold-standard drug used to treat the motor symptoms of Parkinson’s. It is indicated for the treatment of motor fluctuations in advanced PD. DUOPA™ uses the same active ingredients as orally-administered carbidopa/levodopa but is designed to improve absorption and reduce off-times by delivering the drug directly to the small intestine. The procedure carries risks, as does use of the device that delivers the drug. These include movement or dislocation of the tube, infection, redness at the insertion point, pancreatitis, bleeding into the intestines, air or infection in the abdominal cavity and failure of the pump.

### 1.8. A Brief Introduction to Alzheimer’s Disease and Amyloid-β (Aβ)

Another neurodegeneration-related intrinsically disordered protein with great importance of artificial and pathogenic missense point mutations is amyloid-β (Aβ), which is at the center of Alzheimer’s disease (AD). The Aβ peptide is the primary component of extracellular fibrillar deposits, termed amyloid plaques, found post-mortem in brain tissues of patients with AD [78]. Aβ peptides are capable of forming distinct polymorphic structures, ranging from globular oligomers to mature fibrils. Fibrillar structures have been widely investigated through experiments (see, for example, [79,80,81,82]) However, interest has gradually shifted toward smaller oligomers and monomers, as a growing body of evidence points to these structures formed by oligomers, which in turn are formed by monomers, as the pathogenic agents involved at the onset of AD [83,84,85,86,87,88]. The amyloid precursor protein (*APP*) gene encodes for at least four protein isoforms, which are found in various tissues including the central nervous system. Although the normal functions of these proteins are presently not known, at least two isoforms contain functional protease inhibitor domains. In AD, one or more forms of APP is cleaved to yield 39–43 amino acid fragments called Aβ (see [83,84,85,86,87,88] and references therein). This fragment is found in amyloid deposits associated with the cerebral vascular system and with neuritic plaques. Point mutations in the APP located within or in the vicinity of the full-length Aβ have been linked to AD.

Aβ monomer is described as a random coil by solution nuclear magnetic resonance (NMR) and circular dichroism (CD) [89,90]. Due to their heterogeneity and high propensity to aggregate, the low molecular weight Aβ oligomers are not amenable to NMR and X-ray crystallography. As a result, only low resolution structural data from CD, ion mobility mass spectrometry (IM-MS), electron microscopy (EM), transmission electron microscopy (TEM) and atomic force microscopy (AFM) measurements are available [89,90,91,92,93,94,95,96]. At the end of the reaction, the fibrils are insoluble and we are left with complicated experiments using isotopic labeling to propose models. These experiments revealed that fibrils of synthetic Aβ_42_ peptides have U-shaped conformations with β-strands at residues L17–F20 and I31–V40 with the N-terminal residues disordered, while fibrils of synthetic Aβ_40_ peptides have β-strands at Y10–D23 and A30–G38 with the N-terminal residues [79,97,98,99]. Fibrils made of Aβ40 peptides show, however, deformed U-shaped conformations, with a twist in residues F19–D23, a kink at G33 and a bend at G37–G38 and a more ordered N-terminus [100]. Overall, the final products are very sensitive to the nature of the sample (synthetic or brain-derived Aβ peptides). Fibril formation is also under kinetic control rather than thermodynamic control, adding further complexity to the determination of the physical factors governing Aβ fibril formation [101,102]. The amino acid sequence of human Aβ_42_ is shown below:

DAEFRHDSGYEVHHQKLVFFAEDVGSNKGAIIGLMVGGVVIA

The formation of certain secondary structure elements is key to oligomerization and aggregation mechanisms, such as α-helix and β-sheet formation (see [84] and references therein). Blocking residues that adopt abundant α-helix and β-sheet structures can prevent the toxic oligomerization and aggregation processes by using small molecules (drugs) or antibodies [84]. Therefore, understanding the secondary structure and tertiary structure properties along with thermodynamic properties at the atomic level with dynamics helps in designing new drugs and antibodies.

### 1.9. Aβ Mutations and Alzheimer’s Disease

#### Mutations in N-Terminal Region

Artificial mutations are widely utilized to study the structure and function mechanism of the intrinsically disordered Aβ peptide. However, pathogenic missense mutations exist as well. For example, Goate et al. examined the cosegregation of AD and markers along the long arm of chromosome 21 in a single family with AD confirmed by autopsy [103,104]. They demonstrated that in this kindred—which shows linkage to chromosome 21 markers—there was a point mutation in the *APP* gene. This mutation caused an amino acid substitution (Val→Ile) close to the C-terminus of Aβ. Two familial single point mutations were reported in the N-terminus of Aβ as well. A missense mutation in an Italian family, A2V, caused an early onset of AD when it was only inherited from both parents, while heterozygous carriers of A2V were unaffected. It was also shown that A2V enhances Aβ_40_ aggregation kinetics but the mixture of the WT and A2V Aβ_40_ peptides was protective against AD [105]. Another striking result (same residue) came from the analysis of *APP* in a set of whole-genome sequence data from 1795 Icelanders that resulted in the discovery that the A2T mutation is able to protect against AD in both heterozygous and homozygous carriers [106]. The English familial disease mutation (H6R) of Aβ was reported by Janssen et al. [107]. Another single point missense mutation in the N-terminus of Aβ was reported for a Taiwanese family (D7H) [108]. The same 7th residue (D) is also affected by another single point mutation in a Tottori family (D7N), causing early onset familial AD [109,110].

### 1.10. Mutations in the Middle Part of Aβ

Among the various hereditary mutants of Aβ in familial forms of AD, the A21G Flemish-type mutant has unique properties showing a low aggregation propensity but progressive deposition in vascular walls [111,112]. Four of the genetic missense mutations (Italian E22K, Dutch E22Q, Arctic E22G and Iowa D23N) cluster in the region of E22 and D23 in the Aβ sequence and they have higher neurotoxicity compared to the WT Aβ peptide (see [113,114,115] and references therein). These mutations are thought to modify the physicochemical properties of the peptide. For example, kinetic studies show that the E22K and E22Q mutations lead to faster peptide aggregation, whereas the E22G and D23N mutations result in slightly slower aggregation than WT Aβ42 (although the E22G mutation shows increased protofibril formation) [116,117,118,119,120,121,122,123]. Solid-state NMR studies also suggest that rather than the in-register β-sheet conformation adopted by WT Aβ, the Iowa D23N mutant forms amyloid fibrils with antiparallel β-sheet structure (see [124] and references therein).

A rare mutation in APP with a deletion of glutamic acid, ΔE693, was first identified in patients of Japanese pedigree [125]. In clinical studies on propositus, it was discovered that the variant of APP is closely linked to the pathogenesis of AD with symptoms similar to AD-type dementia. The ΔE693 mutation in APP produces a form of Aβ that lacks a Glu22 residue, ΔE22, which is known as the Osaka mutant [125]. Initial studies in vitro and in vivo suggested that the mutant did not form fibrils but presented subcellular oligomers in transfected cells [126]. Subsequent in vivo studies showed that the transgenic mice exhibited age-dependent intraneuronal Aβ oligomerization without extracellular amyloid deposits [127]. In contrast to earlier reports that the Osaka mutant (ΔE22) did not form fibrils, recent studies, however, demonstrated that the mutant Aβ peptides have strong tendencies to form fibrils faster than the WT Aβ (see [128] and references therein). The structural and thermodynamic properties of these mutants were not known until MD simulations were conducted on these species.

### 1.11. Some Approaches to the AD Treatment

Currently available medications cannot cure AD or stop its progression. Available drugs may help in lessening symptoms, such as memory loss and confusion for a limited time. The U.S. Food and Drug Administration (FDA) has approved two types of medications; cholinesterase inhibitors, such as Aricept, Exelon and Razadyne and memantine (Namenda) to treat cognitive symptoms of AD [129,130,131,132]. There is also a medication that combines one of the cholinesterase inhibitors (donepezil) with memantine called Namzaric [133]. Cholinesterase inhibitors prevent the breakdown of acetylcholine), which is a chemical messenger that is crucial for learning and memory. This supports communication between nerve cells by keeping acetylcholine levels high. These delay or slow worsening of symptoms. Side effects include nausea, vomiting, loss of appetite and increased frequency of bowel movements. Among three cholinesterase inhibitors that are commonly prescribed, Aricept is approved to treat all stages of AD whereas Exelon and Razadyne are approved to treat mild to moderate AD [134,135,136]. Memantine regulates the activity of glutamate, which is a chemical involved in information processing, storage and retrieval [137]. Although it improves mental function and ability to perform daily activities, the use of memantine might produce severe side effects, such as headache, constipation, confusion and dizziness [138]. Scientists are developing novel benzopolycyclic amines with increased NMDA receptor antagonist activity and are targeting BACE1 and Tau and Aβ proteins.

### 1.12. Current Challenges in Designing Dugs for PD and AD Treatment

Despite many in vitro and in vivo studies, drug after drug has failed to slow the progression of AD and PD due to the following reasons:

Monomers and oligomers of αS and Aβ are the most critical players in the pathology of PD and AD, respectively and larger aggregate and fibril production are toxic as well, however, there is currently limited information about their formation rates in the patient brain (see [8,84,139,140,141,142] and references therein). Experimental and computational studies showed that these disordered proteins self-assemble into fibrils by a nucleation–condensation polymerization mechanism [143,144,145]. While equations enable interpretation of the experimental sigmoidal kinetic profiles of formation by means of primary and secondary nucleation processes, they do not provide any information on the 3D topology and size of the primary nucleus. Overall, probing the conformational changes of αS and Aβ is challenging due to the intrinsically disordered nature of these proteins, as well as because of the vast heterogeneity of the resulting aggregates, the number of monomers in each aggregate type and the sensitivity of the process to pH, agitation, temperature, concentration, ionic strength, surfactants, sample preparation and the fragment size.

### 1.13. Experimental and Computational Approaches for the Analysis of αS and Aβ Structures

A full understanding of PD and AD (as well as related to them intrinsically disordered proteins αS and Aβ) requires the development and use of innovative biophysical techniques. Along with standard approaches, e.g., Fourier transform infrared spectroscopy (FTIR), CD, X-ray powder diffraction, TEM, AFM, solid state nuclear magnetic resonance (ss-NMR), dynamic light scattering (DLS) and IM-MS, new techniques are being applied. These include, notably, pulsed hydrogen/deuterium exchange coupled with mass spectrometry analysis, which unlike fluorescence methods, does not require labeling with a fluorophore, photonic crystal-based approaches, single molecule imaging techniques and specific isotope labeling with electron paramagnetic resonance (EPR), advanced hyperfine sublevel correlation (HYSCORE) and electron–nuclear double resonance (ENDOR) methods [146,147,148,149,150,151,152].

Experimental studies alone are not sufficient for producing a clear picture, since they usually yield time- and space-averaged structural and thermodynamic properties. Molecular dynamics (MD) simulations by exploring different time and length scales at the atomic level complement experiments [153,154,155]. MD simulations are very challenging due to the inherent flexibility of heterogeneous ensemble of the αS and Aβ monomers and oligomers and the impact of artificial or genetic mutations on the structures and thermodynamic properties of αS and Aβ in PD and AD. Computer-aided drug design that focuses on searching for potential inhibitors for the formation of αS and Aβ fibrils and aggregates is of great interest [156,157,158,159,160,161,162,163,164,165,166]. In his remarks at the Regulatory Affairs Professionals Society’s (RAPS) 2017 Regulatory Convergence Conference, U.S. Food and Drug Administration (FDA) Commissioner Dr. Scott Gottlieb, spent considerable time addressing how “seamless” clinical trials and more widespread use of modeling and simulation could help combat the costs of both drug development and new drugs (Available online: https://www.fda.gov/NewsEvents/Speeches/ucm575400.htm). As part of an effort to advance use of MD tools, the Agency plans to convene a series of workshops, publish guidance documents, develop policies and procedures for translating computational approaches into regulatory review and conduct pilot programs on these approaches.

Development of the inhibitors (drugs) of fibril and aggregate formation requires understanding of the structures and thermodynamic properties of monomeric and oligomeric forms of αS and Aβ, as well as elucidation of the impacts of mutations on structures of these IDPs at the atomic level with dynamics. MD simulation techniques provide a useful tool for investigating these disordered monomeric and oligomeric structures in solution with dynamics at the atomic level. Over the last decade, enormous progress has been made on recording the health state of an individual patient down to the molecular level of gene activity and genomic information. In fact, sequencing a patient’s genome for less than 1000 dollars is no longer an unrealistic goal. However, the ultimate goal is to use all this information for personalized medicine that is to tailor medical treatment to the needs of an individual, remains largely unfulfilled. Despite the rich potential of MD simulations in personalized medicine, its impact on data-driven medicine remains low, due to a lack of experts with the knowledge in both drug synthesis and in molecular dynamics simulations.

We provide here an in-depth review on the contribution of MD simulations to characterize the molecular structures of αS and Aβ in solution. We focus on the impact of artificial and genetic missense mutations on αS and Aβ in solution at the atomic level with dynamics. We then conclude by offering a perspective on the future of the field along with MD simulations and the major questions that need to be addressed to discover drugs with much higher efficacy.

## 2. Artificial and Pathological Mutations in α-Synuclein: Insights from Molecular Dynamics Simulations

Characterizing the monomeric state of αS in atomic detail under physiological conditions can be a key to understanding how αS assembles into disease-causing oligomers because they represent a base state common to all fibrillation and aggregation pathways. This knowledge could be crucial for the development of therapeutics that prevent nontoxic monomers from progressing into toxic species, one of the fundamental strategies in the ongoing effort to treat PD (see above). It is well established that self-assembly is profoundly influenced by missense mutations. The polymorphism of monomeric αS under physiological conditions may underlie this relationship. In the absence of unambiguous stable native states, simple chemical modifications could have a profound effect on the type of ensemble sampled by αS protein [167,168,169,170,171,172,173,174]. In addition to high aggregation propensity of αS, the intrinsically disordered nature has frustrated experimental efforts to characterize the 3D structures of this protein with dynamics at the atomic level [175].

The challenges and limitations inherent to the current set of experimental techniques for studying the intrinsically disordered, aggregation prone αS monomers have encouraged some groups to use MD simulations to more thoroughly investigate the conformational properties of this IDP (see, for example, [176,177,178,179,180]). Simulations for αS extend over multiple microseconds. In addition, replica exchange molecular dynamics simulations (REMD), simulated tempering are utilized to escape energy minima and enhance sampling [181,182,183,184,185,186,187]. The results obtained from simulations of IDPs such as αS and Aβ strongly depend on the set of force field parameters used to describe the energy of an IDP and its interactions with the aqueous solvent [188,189,190,191,192]. Widely used force field parameters are AMBER FF99SB and its variants, CHARMM22/CMAP, OPLS-AA while implicit or explicit models for water are utilized [188,189,190,191,192]. These force field parameters have been calibrated against model compounds and peptides and in most cases, the force field reproduce folded conformations of small globular proteins with root-mean-square deviations (RMSDs) within angstroms of the experimentally determined structures. However, experimental validation of the ensembles obtained using these force field parameters for IDPs remains an unsolved problem. Here, we review some of the more recent simulation studies, which employ state-of-the-art strategies to characterize the equilibrium structures of the WT and mutant αS in aqueous solution under physiological conditions at the atomic level with dynamics.

The highly acidic C-terminal region of human αS contains three of the four Tyr residues at positions 125, 133 and 136. The fourth Tyr is located in the N-terminal region at position 39. It was proposed that interactions between the C-terminus and the central portion of this IDP may prevent its fibrillation/aggregation [193,194]. NMR studies showed that αS adopts an ensemble of conformations that are stabilized by long-range interactions [193]. In particular, a long-range intra-molecular interaction between the C-terminal region (residues 120–140) and the central part of α-synuclein (residues 30–100) was noted. This interaction was proposed to inhibit fibrillation and could arise from electrostatic or hydrophobic or both types of interactions. If hydrophobic interactions are important, then the cluster of three Tyr residues in the C-terminus is likely to play an important role in aggregation and fibrillation of this protein. To test this hypothesis, Fink and co-workers examined the roles of Tyr residues using artificial mutations (Tyr→Ala) on the αS propensity to fibrillate using various experiments, including thioflavin T (ThT) fluorescence assay, FTIR and CD measurements [195]. They reported that fibril formation of αS was inhibited by substituting the three C-terminal Tyr residues with Ala. Substitution of Tyr133 by Ala resulted in the absence of fibrillation, whereas Y125A and Y136A mutants showed limited inhibition of the fibrillation process. Structural analysis revealed that the Y133A mutant had a substantially different conformation rich in α-helical structure, as compared with the WT αS and its other mutants [195,196,197]. However, the formation of tertiary structure could not be observed in near-UV CD spectra.

Mattaparthi and co-workers used all-atom MD simulations and investigated the conformational dynamics of WT αS and its three Tyr mutants (Y39A, Y133A and triple mutant Y125A/Y133A/Y136A) [198]. They conducted MD simulations for 30 ns using the AMBER FF99SB force field parameters for the wild type and mutant αS and an implicit solvent model for water (generalized Born, GB) [198]. Among the WT and the mutants analyzed, they observed Y125A/Y133A/Y136A and Y133A to have lesser number of hydrophobic contacts between the residues in the N- and C-terminal regions, exhibiting a different folding pattern and conformation that has the ability to delay the aggregation of αS. Even though their simulation was short and they did not use special sampling methods, they reported an increase in the helical structure content, reduction in the β-sheet content and different conformational stability for the artificial mutants [198]. They also found that Tyr residue at position 133 is primarily important to drive the intramolecular interactions and subsequent fibrillation process of αS. Although their mutation studies using MD simulations might help in better understanding of the conformational behavior of αS in aqueous solution, the simulation time should be longer and special sampling techniques should be utilized to generate more reliable data.

Coskuner and co-workers studied the structure of the WT αS and the impacts of A53T, E46K and A30P pathological missense mutations on the structure of this protein [199,200,201]. IDPs can adopt a multitude of different conformations. As a result, the theoretical method for investigating IDPs needs to be chosen carefully, so that the different possible protein conformations are adequately sampled. REMD simulations utilize special sampling throughout the course of the simulation to overcome energy barriers between different conformations with minimal energy [202,203]. Coskuner and Wise-Scira performed extensive REMD simulations utilizing the AMBER FF99SB force field parameters for the wild type and mutant proteins [199]. The usage of an explicit solvent model in REMD simulations can result in errors due to variations in the heat capacity of water, as well as conformational effects due to confined aqueous volume effects. Therefore, the Onufriev-Bashford-Case generalized born implicit solvent model was utilized in these simulations [188,204]. A total number of 56 replicas were employed for the WT αS and its A53T mutant, with temperature exponentially distributed between 283 and 400 K [199], yielding an exchange probability of 0.70 [202,205]. Langevin dynamics were used to maintain the temperature of each replica with a collision frequency of 2 ps^−1^ [206,207,208]. The bonds to hydrogen atoms were constrained using the SHAKE algorithm. Despite the confined aqueous volume effect in the simulations of highly flexible large-size IDPs, usage of an implicit water model ignores the impact of inter-molecular hydrogen bonding interactions as well as short- and long-range solvent structuring and local density effects on the determined IDP conformations. Therefore, sets of additional simulations were conducted utilizing specific WT and mutant conformations that were obtained from their REMD simulations using an implicit water model as the initial structures. All structures were solvated using the modified TIP5P model for water in a box where the closest distance between the protein and any box edge was 20 Å and simulated for additional 30 ns via separate classical MD simulation runs at the same temperature and pressure of interest (temperature of 310 K and pressure of 0.1 MPa) [209,210]. The cumulative secondary structure abundance was used to verify the convergence of the REMD simulations of the WT and A53T mutant proteins at 20 ns of simulation time. The structural and thermodynamic properties of the WT and A53T mutant proteins were calculated from the structures obtained after convergence from the replica closest to physiological temperature (310 K) [199]. The abundances of the secondary structure components per residue for the WT and A53T mutant proteins were calculated via the DSSP program [211]. Additionally, they applied their own theoretical strategy to calculate the free energy change associated with transitions between two different secondary structure components at the atomic level with dynamics [199,200,201,212]. This method calculates the potential of mean force (PMF) of each transition via the conditional probability, defined as $P\left(t_{i\to j}|S_{t_{j}}\right)$. Within this conditional probability, $P\left(t_{i\to j}\right)$ is the probability of a transition between two different secondary structure, *i* and *j*, while $P\left(S_{t_{j}}\right)$ identifies the probability of a transition resulting in the formation of a specific secondary structure, *j*, for a certain residue [199,200,201,212]. The free energy change of each secondary structure transition is then calculated using the ProtMet software package (Equation (1)):

(1) $$PMF=-k_{B}\;T\ln Z\left(\lambda \right)$$

where *k_B_* is the Boltzmann constant, *T* is the temperature, λ is the interconversion probability, and *Z* is the conditional probability ratio of the specific secondary structure transition. More details can be found in [199,200,201,212]. The intramolecular interactions in the WT and A53T proteins were determined by calculating the probability of interactions between two different residues. Intramolecular interactions between two different residues occur if a heavy atom (C, N, O, or S) of a residue is at least 20 Å from a heavy atom of any other residue. The thermodynamic preferences of the WT and A53T mutant proteins were determined using both the MM/PBSA and PMF methods [199,200,201,212,213]. The MM/PBSA method utilizes the potential energy (*E_tot_*), solvation free energy (*G_sol_*) and entropy (*S*) of each protein structure to calculate the estimated conformational Gibbs free energy (*G*) of the same protein structure at a specific temperature (T) via Equation (2):

(2) $$G=E_{tot}+G_{sol}-TS$$

The *G_sol_* is the summation of the electrostatic and nonpolar contributions of each protein structure to the *G*. The electrostatic contribution to the *G* is calculated using dielectric constant values of 1 and 80 for the protein and solvent environment, respectively. The entropy values were estimated using the normal-mode analysis method [214]. Entropy value calculations using a quasi-harmonic method, namely, the Schlitter method, were also attempted. However, the conformational changes were too large for this method to be applied [215]. The coordinates of *R*_g_ and *R*_E__-E_ were used to determine the PMF surfaces of both the WT αS and its A53T mutant.

**Figure 1:**
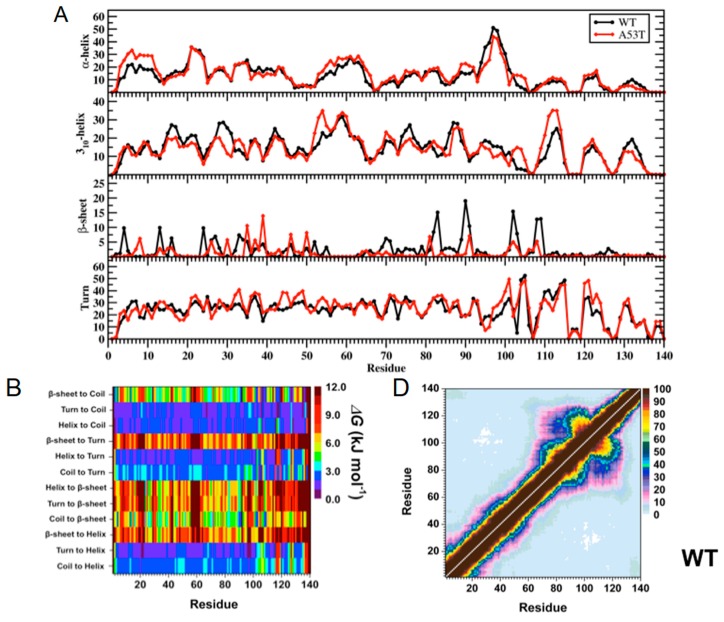

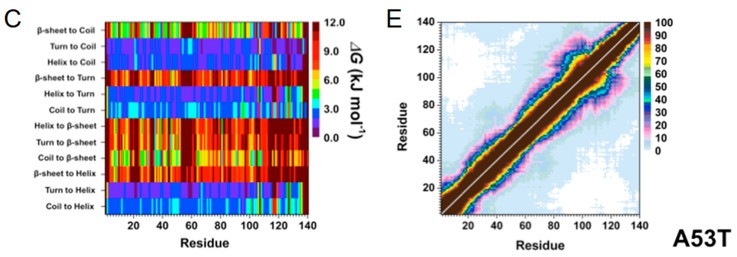
(**A**) WT and A53T Mutant αS Secondary Structure Components. Secondary structure component abundances per residue for the WT (black) and A53T mutant (red) αS structures obtained after convergence. The abundances for the π-helix and coil structures are not displayed; (**B**) WT and A53T Mutant αS Secondary Structure Transition Stabilities. The stability of secondary structure transitions between two specific secondary structure components per residue for the WT (WT) and A53T mutant (A53T) αS proteins based on free energy calculations performed using a recently developed theoretical strategy; TISS. The color scale corresponds to the free energy value associated with the specific secondary structure transition between two secondary structure components for a specific residue; (**C**) WT and A53T Mutant αS Tertiary Structures. Calculated intra-molecular interactions of the WT (WT) and the A53T mutant (A53T) αS. The color scale corresponds to the probability (P) of the distance between the heavy atoms (C, N, O, S) of a residue being ≤20 Å from each other; (**D**) the tertiary structure map for the WT αS and (**E**) the tertiary structure map for the A53T mutant αS.

Overall, the structural and thermodynamic properties including the conformational Gibbs free energies and secondary structure conversion free energies at the atomic level with dynamics were reported for the WT and A53T mutant in aqueous solution. This analysis revealed the impact of the A53T mutation at the monomeric level on the αS protein structure and dynamics [199]. Even though some structural properties have been before described based on the experimental and theoretical analyses [16,21,53,54,55,141,216,217,218,219,220,221,222,223,224,225,226,227,228,229,230,231,232,233], in this work, all structural properties were presented in detail along with the thermodynamic properties. The secondary structure elements from this work are shown in Figure 1A. Specific secondary structure components, such as α-helix and β-sheet structures, are proposed to play important roles in the physiological function and aggregation mechanism of the αS protein [199]. The helical content of the WT αS was minimally affected by the A53T mutation, except for a few residues in the N-terminal and C-terminal regions. This result agreed with the CD measurements that reported similar α-helical contents for the WT and A53T αS proteins [54,55,226,233]. In addition, these findings supported the findings of Bussel and Elizier, who revealed that the α-helical character of Ala18–Gly31 is unperturbed by the A53T mutation via NMR measurements [16].

Furthermore, Coskuner and Wise-Scira observed that the abundance of the α-helical structure was greater in the N-terminal and NAC regions than in the C-terminal region, especially for the last 38 residues, for both the WT and A53T mutant in aqueous solution [199]. This finding agreed with the observed helical tendency of the first 100 residues of the WT and A53T αS proteins via NMR measurements. Johnsson et al. also detected the same trend for the WT αS structures via Monte Carlo simulations [234]. We should mention here that the formation of α-helical structure in the N-terminal and NAC regions has been proposed to be a key factor for the vesicle and membrane binding [11,16,221,222,235,236,237]. Therefore, the overall similarity in the α-helical contents in the N-terminal and NAC regions of the WT and A53T αS proteins indicates that the binding of αS to vesicles and membranes would not be significantly influenced by the A53T mutation [199]. In fact, several in vitro and in vivo experiments reported that the binding affinity of αS with cell membranes and phospholipid vesicles is unaffected by the A53T mutation [16,217,220,221,222].

Prominent β-sheet formation occurs at residues Leu8, Ala30, Glu35, Val37, Tyr39, Glu46 and His50 in the A53T structures in comparison to those of the WT αS protein [199]. Overall, these findings show an increase in β-sheet formation close to the mutation site in the N-terminal region upon A53T mutation. These results, at the atomic level, with dynamics in aqueous solution support the findings of Bussell and Eliezer, who reported more likely β-sheet structure formation around the mutation site of the A53T mutant in comparison to the WT protein via NMR measurements [231]. However, through their REMD simulations, Coskuner and co-workers also presented the specific residues along with the probabilities of the secondary structure components [199]. Abundant β-sheet structure upon A53T mutation was also observed for αS via single molecule force (SMF) and Fourier transform infrared (FTIR) spectroscopy measurements [54,55,227]. Interestingly, Balesh et al. did not detect an increase in β-sheet propensity around the mutation site of the A53T mutant in comparison to the WT protein via annealing MD simulations [238]. The formation of β-sheet structure in αS has been linked to its aggregation process [54,55,227]. Therefore, results of REMD simulations demonstrated that some specific residues located in the N-terminal region around the mutation site (Ala30–His50) may play an important role in attenuating the aggregation mechanism of αS due to the increase in β-sheet content upon A53T mutation (Figure 1A,B) [199].

Using REMD simulations, Coskuner and co-workers reported also the effect of A53T mutation on tertiary structure properties of αS in aqueous solution at the atomic level with dynamics [199]. Based on these findings (Figure 1C), Gly86–Asn103 and Glu104–Asn122 located in the NAC and C-terminal regions present strong intramolecular interactions (>50%) in the WT αS structures in aqueous solution (Figure 1D). Additionally, stable intramolecular interactions (up to 88%) within the NAC region of the WT αS are detected between Val70–Gly84 and Ala85–Leu100. Furthermore, abundant intramolecular interactions occur between Ala56–Gly106 and Gly84–Gln134 (up to 42%). Upon A53T mutation, the abundance of intramolecular interactions between the NAC and C-terminal regions (Gly86–Asn103 with Glu104–Asn122) and within the NAC region (Val70–Gly84 with Ala85–Leu100) decreases (Figure 1E) [199]. Furthermore, the intramolecular interactions between Ala56–Gly106 and Gly84–Gln134 are reduced (<20%) as a result of the A53T mutation. The abundance of interactions between Glu28–Glu46 in the N-terminal region and Glu60–Lys80 in the NAC region, as well as within the C-terminal region between Gly86–Glu104 and Glu130–Ala140, increases slightly upon A53T mutation. It was also reported that the intramolecular interactions of the C-terminal region with the N-terminal or NAC regions almost disappear upon A53T mutation [199].

Carloni and co-workers reported a similar loss in intramolecular interactions caused by the A53T in αS protein using classical MD simulations in explicit water [239]. Furthermore, NMR measurements of the WT and A53T mutant performed by Bertoncini et al. also reported decreased long-range interactions upon A53T mutation, especially between the C-terminal and NAC regions [21]. Therefore, these studies presented the reduced long-range interactions involving the NAC region and indicated that the NAC region is more solvent-exposed upon A53T mutation. This hypothesis was further supported by Hazy et al. who reported that the A53T mutant is more hydrated than the WT αS via differential scanning calorimetry measurements [240]. Increased exposure of the NAC region is related to the enhanced aggregation propensity of αS due to the proposed critical role of this region in the aggregation process [53,54,55,223,224,226,229,230,232,233]. Therefore, tertiary structure findings using REMD simulations indicate that the aggregation propensity of the αS protein is increased upon A53T mutation, which is in agreement with previous experiments [199].

Dobson and co-workers used 101 αS structures in their MD simulations, which were determined by a combination of paramagnetic relaxation enhancement NMR spectroscopy and ensemble MD simulations as the best structural approximation of the disordered state of αS [24]. For comparison, 10 monomeric globular structures (of which nine—PDB codes 1E20, 1E9H, 1NB0, 1NUN, 1P5V, 1PK6, 1USU, 1XGW and 1Z2F—were comparable in size to αS and one—1NDD—was an ubiquitin like structure) were also selected. For modeling of the hydration shell, they used the solvate Shell function of the sleap program (AmberTools 1.4 http://ambermd.org/) with a shell thickness of 2–8 Å (force field, leaprc.ff03.r1; water model, TIP3PBOX). After MD simulation, the PERL scripts were used to calculate the number of water molecules in the hydration shell by calculating, for each water molecule, the distance between the water molecule (oxygen atom) and the nearest heavy atom of the protein and determining the number of water molecules within a given distance range. Their simulation results showed that the A53T mutant of αS displayed a higher level of hydration than the WT αS, suggesting a bias to more open structures, favorable for protein-protein interactions leading to amyloid formation. These differences disappeared in the amyloid state, suggesting the same surface topology, irrespective of the initial monomeric state [24].

REMD simulations conducted on pathological A30P missense mutation revealed that within the N-terminal region (Met1–Lys60) of the WT αS and its A30P mutant, there was the abundant α-helix formation at Ala19–Lys23 and the 3_10_-helix formation at Val15–Ala18, Glu20–Thr22, Gly41–Thr44 and Thr54–Lys60 varying between 20% and 35% [201]. Interestingly, Gly7–Glu13, Val15–Ala17, Lys32–Val40 and Lys43–Gly47 adopted more prominent helical structure (α-helix or 3_10_-helix; up to 30%) in the structures of the A30P mutant than in those of the WT αS protein. In contrast, the α-helix and 3_10_-helix contents at Gly25–Lys32 in the WT αS protein structures decreased or disappeared as a result of the A30P mutation. This finding is in agreement with the NMR measurements that reported reduced helical propensity for Ala18–Gly31 upon A30P mutation [231,241]. Chatterjee and Sengupta also presented a decrease in helix abundance around the mutation site of the A30P mutant of αS in comparison to the WT protein by conducting MD simulations [242]. Bussell and Eliezer proposed that destabilization of helix formation in this region may be associated with the increased rate of oligomerization of the A30P mutant rather than WT αS [231]. Consequently, REMD simulation results suggested an increase in oligomerization rate of the A30P mutant in comparison to the WT protein. Abundant β-sheet structures (5% to 20%) were formed in parts of the NAC and C-terminal regions (Val70, Val71, Val82, Glu83, Ala89–Ala91, Lys102, Asn103, Pro108 and Gln109) in the structures of the WT αS protein [201]. A similar trend was observed for the A30P mutant structures, with a significant increase in β-sheet formation at Val66, Gly67, Ile88, Ala89, Val95–Gln99, Gly101, Lys102 and Pro108–Glu114. These REMD simulation findings support previous NMR measurements that reported β-sheet structure in the C-terminal region of both the WT and A30P mutant forms of αS [231]. Furthermore, SMF and FTIR measurements reported increased β-sheet conformation for the A30P mutant structures in comparison to those of the WT αS structures [54,55,243]. In contrast to these REMD simulation findings, annealing MD simulations performed by Balesh et al. did not show an increase in β-sheet formation in the A30P mutant structures in comparison to those of the WT protein [238]. Additionally, Coskuner and co-workers found that the N-terminal region possessed abundant β-sheet structure (≥5% probability) at Phe4, Glu13, Val16, Gln24, Val26, Ala27, Thr33–Glu35 and Val37 in the WT αS conformations that disappeared in the structures of the A30P mutant protein [201]. This result supported the NMR measurements performed by Bussell and Eliezer, who suggested a possible decrease in the β-sheet formation upon A30P mutation in the N-terminal region of the WT αS protein [231]. As aforementioned, β-sheet structure formation has been linked to the aggregation process. Therefore, the N-terminal region of A30P mutant is less likely to participate in the aggregation process than the same region in the WT αS. Furthermore, REMD simulation results predicted that the C-terminal region and part of the NAC region of A30P mutant are more reactive toward aggregation than the same regions in the WT protein [201].

These findings support the experimental findings reporting a faster rate of oligomer formation for the A30P mutant in comparison to the WT αS [53,54,55,141,229,230,232,233]. We should mention here again that the β-sheet formation is associated with the self-association and aggregation of αS, including the formation of dimeric, oligomeric and fibril structures [53,54,55]. Furthermore, β-sheet formation in the NAC region was proposed to play an important role in the intermolecular interactions between the monomeric species [231]. Therefore, the increased β-sheet structure formation in the NAC and C-terminal regions found upon A30P mutation may be associated with the reported higher oligomerization rates detected for the A30P mutant. Coskuner and co-workers detected strong intramolecular interactions between Gly86–Asn103 in the NAC region and Glu104–Asn122 in the C-terminal region with an abundance larger than 50% in the structures of the WT αS (see above) [201]. Furthermore, Val70–Gly84 and Ala85–Leu100 in the NAC region of the WT protein present strong intramolecular interactions (up to 90%). Prominent interactions occurred between Ala56–Gly106 and Gly84–Gln134 (up to 40%). Therefore, moderate interactions were found between the N-terminal, NAC and C-terminal regions with the NAC and C-terminal regions. Overall, these tertiary structure findings agree with the previous theoretical studies performed by Carloni and co-workers [239].

Interestingly, the intramolecular interactions in the WT αS structure are significantly influenced by the A30P mutation. Even though some intramolecular interactions between a part of the NAC region (Ile88–Asn103) and the C-terminal region (Glu104–Pro120) occur, the abundances of these interactions are decreased in the A30P mutant. Furthermore, abundant (up to 40%) intramolecular interactions occur between Lys58–Val95 and the C-terminal region (Lys96–Pro128) [201]. Intramolecular interactions between the N-terminal region (Val26–Lys58) and the NAC and C-terminal regions (Gln62–Leu100) were also detected. However, interactions between the N-terminal region (Met1–Lys60) and Val118–Val140 of the C-terminal region disappeared upon A30P mutation. Similar trends were also detected for the intramolecular interactions between the residues Met1–Val16 of the N-terminal region and the NAC region. This finding along with the decreased intramolecular interactions between the NAC and C-terminal regions of the A30P mutant αS suggested that the NAC region is more solvent exposed in the A30P mutant as compared with the structures of the WT αS [201].

The reduced long-range intramolecular interactions as well as the increased exposure of the NAC region upon A30P mutation agree with some previous NMR measurements [231,239]. Furthermore, the less abundant long-range interactions and increased exposure of the NAC region have been proposed to potentiate the aggregation of the WT αS by allowing the NAC region, which is proposed to be a key in the fibrillogenesis process, to be more available for intermolecular interactions with surrounding monomers rather than intramolecular interactions [21,241,244]. Therefore, these tertiary structure findings along with investigations of secondary structure propensity suggested that the A30P mutant of αS tends to be more reactive toward aggregation than the WT αS, which is in agreement with some experimental data [53,54,55,229,230]. Time-resolved fluorescence energy transfer measurements reported an increased donor to acceptor distance upon A30P mutation of αS, which agrees with their less compact structure of A30P mutant αS in comparison to the WT αS [201,245].

**Figure 2:**
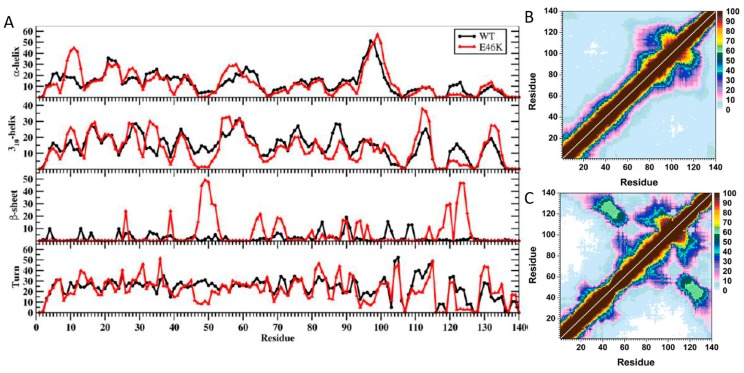
(**A**) WT and E46K αS secondary structure components. Secondary structure abundances per residue for the WT (black) and E46K mutant (red) αS. The abundances for the π-helix and coil structures are not displayed; (**B**,**C**) WT and E46K αS tertiary structures. Calculated intramolecular interactions of the WT (WT) and the E46K mutant type (E46K) αS. The color scale corresponds to the probability (P) of the distance between the heavy atoms (C, N, O, S) of a residue being ≤20 Å from each other.

The impact of E46K pathological missense mutation on the structures and thermodynamic properties of the αS protein in an aqueous solution was also investigated using REMD [200]. The most abundant α-helix formation in the N-terminal region of the WT αS occurs in the Glu20–Gln24 region with 20–35% abundance (Figure 2A). Even though the abundance of α-helical structure of this Glu20–Gln24 region was similar in the E46K mutant of αS, the most abundant α-helical structure in the N-terminal region of the E46K mutant was detected at the Ser9–Glu13 region, which was at least 15% more abundant than in the WT αS (Figure 2A) [200]. Furthermore, Ala27–Ala29 and Thr54–Thr59 regions also presented an increase in the α-helical structure upon the E46K mutation in αS. However, the opposite trend was observed for residues Tyr39–Ser42. In the non-amyloid β component region (NAC; Glu61–Val95), residues Glu61–Thr64 of the WT αS formed abundant α-helical structure. The formation of helical structure in the N-terminal and NAC regions was associated with the lipid or vesicle binding of the αS [11,221,236,246,247]. Therefore, differences in the α-helix formation of the αS as a result of the E46K mutation may affect the binding of this protein with lipids or vesicles. Previous experimental studies reported that the E46K mutant of αS has a higher affinity for binding to negatively charged vesicles than the WT protein [217]. The REMD simulation results supported these observations and further showed that reported higher affinity may be associated with the higher α-helical content of the Ser9–Glu13, Ala27–Ala29 and Thr54–Thr59 regions in the N-terminal region caused by the E46K mutation (Figure 2A) [200]. Within the C-terminal region (Lys96–Ala140), the most prominent α-helix formation happened at residues Lys96–Gly101 for both WT αS and its E46K mutant. However, the abundance of helical structure at Gln99–Lys101 increases by up to 20% in E46K mutant. In addition, the α-helical structure in the Pro120–Glu123 region almost disappeared upon E46K mutation. For the WT αS, the most abundant β-sheet elements (up to 20%) were formed in parts of the NAC and C-terminal regions (Val70, Val71, Val82, Glu83, Ala89–Ala91, Lys102, Asn103, Pro108 and Gln109). It was also observed that the β-sheet structure is formed in the N-terminal region of the WT αS at Phe4, Glu13, Val16, Gln24, Val26, Ala27, Thr33–Glu35, Val37 and Val52 (Figure 2A) [200].

Interestingly, the NAC and N-terminal residues (except Phe4) are located in regions considered as parts of the 11-mer repeats. There are seven regions of 11-mer repeats, sequences that might assume right-handed coiled coil conformations in the N-terminal and NAC region of α-synuclein [248]. It is suggested that these repeats lowered the propensity of α-synuclein to form β-sheet due to their preference for α-helix formation [248]. In comparison, the most prominent β-sheet structures (20–50%) for the E46K mutant were found in the N-terminal and C-terminal regions, at residues Val26, Tyr39 and Gly47–Ala53 and Asp119, Pro120 and Asn122–Tyr125, respectively (Figure 2A). In addition, Val63–Val66, Ala69–Thr72, Lys80, Gly93, Phe94, Lys96, Glu114, Pro117 and Val118 in the NAC and C-terminal regions showed the β-sheet structure formation with 5–20% abundances. Kessler et al. reported that αS was more fibrillogenic upon deletion of the N-terminal and C-terminal regions, thereby implying that the NAC region is more prone to aggregation than other parts of the protein [248]. In comparison with the REMD simulation results, the NAC region had minor β-sheet structure formation (5–20% abundance), whereas the N-terminal and C-terminal regions exhibited a 20–50% abundance of β-structure. Furthermore, specific residues (Val37–Lys43, Val52–Thr59, Gln62–Val66, Gly68–Val77 and Ala90–Val95) were reported in NMR study conducted by Vilar et al. as the main regions to form β-structure that composed the WT αS fibrils [249].

The most abundant (up to 55%) turn structure formations occurred in the C-terminal region of the WT protein but this was shifted to the N-terminal region in the E46K mutant [200]. Large discrepancies in the tendencies to form turn structure in the N-terminal region were noticed at Glu13, Gly14, Ala30, Gly31, Gly36 and Lys46–His50; in the NAC region at Val82, Ala85 and Gly86; and in the C-terminal region at Glu110–Ile112, Pro117, Val118, Asn122–Ala124 and Glu130 and Glu131 with a difference up to 20% (Figure 2A).

Strong intramolecular interactions between the NAC and C-terminal regions of the E46K mutant were also observed between residues Val82–Asp98 and Glu104–Ala124 (Figure 2B,C). In addition, an increase in the moderately abundant interactions (≤50%) between Val70–Val82 and Glu110–Val118 as well as between Val82–Glu105 and Glu126–Ala140 occurred upon E46K mutation [200]. Both, the WT αS and E46K mutant presented strong intramolecular interactions, with a high abundance between residues Val70–Gly84 and Ala85–Leu100 within the NAC region [200]. The E46K mutation also resulted in an increase in the interactions between Val48–Gly67 and Val82–Lys102 by up to 40%. Furthermore, intramolecular interactions between the N-terminal and NAC regions (Gly7–Val66) with the C-terminal region (Gly106–Ala140) were up to 50% more abundant in the E46K mutant than in the WT αS [200]. In agreement with these findings, Rospigliosi et al. presented that interactions between the C-terminal region with the NAC and N-terminal regions were enhanced upon E46K mutation of αS [250]. Interactions within the N-terminal region (e.g., between residues Val16–Ala30 and Gly36–Lys58) were also increased in the E46K mutant in comparison to the WT αS [250]. However, the weak intramolecular interactions (<10%) between the N-terminal region (Met1–Glu20) with the NAC and C-terminal regions (Thr64–Pro120) completely disappear as a result of the E46K mutation [200].

Interestingly, interactions between this N-terminal region (Met1–Glu20) and the NAC region have been used to identify the two different possible vesicle binding structures [243]. Experimental measurements have proposed that the αS binds to vesicle and/or lipid structures in either a broken or extended helical conformation that can depend on the structure of the vesicle or lipid as well as on the protein conformation [243]. Specifically, the extended helical structure is reported to more likely bind to a flat micellar surface, whereas the broken helix structure is more likely to bind to a spherical micellar structure [243]. The broken helical structure presents a distance of ~34 Å between the N-terminal and NAC regions, whereas this distance increases to ~67 Å in the extended helical conformation. The reported simulation results for the interactions between the NAC and N-terminal regions indicated that, upon E46K mutation, the distance was increased and therefore an extended conformation was more likely to form in the E46K mutant rather than in the WT αS [200]. As a result, it was predicted that the E46K mutant may show a higher binding propensity to the flat micellar surfaces than the WT protein, whereas the opposite trend was expected for the spherical micellar surfaces [200].

The average conformational Gibbs free energies along with their enthalpic and entropic contributions for the WT αS and its various mutants are presented in Table 1 [199,200,201]. The structures of the A53T α-synuclein are thermodynamically more preferred than the structures of the WT protein by a ΔG value of 132.9 kJ·mol^−1^. This suggests that the A53T mutant is characterized more stable structure than the WT αS protein. Specifically, the thermodynamic results suggest that the A53T mutant structure is more stable than its WT form largely due to the enthalpic contribution. Overall, these findings can be attributed to the solvent exposure variations of the two IDPs. Based on the difference in conformational Gibbs free energy values, the structures of the WT αS are by 79.4 kJ·mol^−1^ more stable than structures of the A30P mutant in an aqueous medium. The less stable A30P mutant structures might be more prone for aggregation than the structures of the WT αS based on these Gibbs free energy calculations. Therefore, these thermodynamic results supported structural findings described above as well as some experiments, which predicted the increased oligomerization and aggregation rates of the A30P mutant.

Comparing the enthalpic (see above), the increased retention time of the E46K mutant αS in comparison to the WT Protein and entropic contributions to the conformational Gibbs free energies revealed that the WT αS is enthalpically more preferred than the A30P mutant by 96 kJ·mol^−1^, whereas an opposite trend was observed for the entropic contribution (13.5 kJ·mol^−1^). In agreement with the tertiary structure properties obtained using REMD simulations in size-exclusion chromatography (SEC) suggested that the conformations of this mutant are more compact than those of the WT αS. Overall, these findings suggested that the E46K mutant αS structures possess a greater number of intramolecular interactions and are more stable than the WT αS structures in aqueous solution.

In order to quantitatively assess the relative stability of the WT and the E46K mutant structures, conformational Gibbs free energy calculations were performed. The difference between these Gibbs free energy values yielded an estimate of the degree of difference in the stability of the two protein structures. The average thermodynamic values presented in Table 1 indicated that the conformations of the WT αS were thermodynamically more stable over the structures of the E46K mutant by 114.5 kJ mol^−1^. Additionally, the entropic (TS) and enthalpic (H) contributions to the conformational Gibbs free energies indicated that WT αS was entropically and enthalpically more preferred than the E46K mutant by 52.5 and 61.8 kJ mol^−1^, respectively. The decreased thermodynamic preference of the E46K mutant structures in comparison to the WT αS conformations indicated that the aggregation rate of the αS should increase upon E46K mutation. This hypothesis is in agreement with the results of experimental studies that also reported an increased aggregation rate of the E46K mutant in comparison to the WT αS [199,200,201].

Sanjeev and Mattaparthi used micelle-bound 3D structure of the human WT αS (PDB ID: 1XQ8) as an initial structure for their MD simulations without using special sampling techniques [251]. The 3D structures were constructed from the WT structure by replacing His with Gln and Gly with Asp at positions 50 and 51, respectively, using the Swiss-Pdb viewer software. They then conducted explicit MD simulations on WT, H50Q and G51D mutant of αS using the AMBER force field (ff99SBildn) for proteins and the TIP3P water model. For treating the long range electrostatic interactions, Particle-Mesh Ewald (PME) method was used with the default parameters. For non-bonded interactions, the cutoff value was set to 9 Å. After reaching the target temperature, the systems were equilibrated for a time period of 500 ps and then the production run proceeded for 90 ns in the isothermal-isobaric ensemble. Production run was conducted using the Berendsen barostat with a collision frequency and pressure relaxation time of 2 ps and 1 ps respectively [251]. Free energy calculations were conducted using the MM/PBSA method (see above). The presence of anti-parallel and parallel β-sheets was noticed in both the mutants (H50Q and G51D) of αS. However, the anti-parallel β-strands were seen to be higher in G51D mutant when compared to the H50Q mutant and the parallel β-strands to be more prominent in H50Q. Apart from anti-parallel β-strands, it was noticed that the helical contents were higher in the G51D mutant in comparison with the H50Q mutant. Therefore, from the probable secondary structure analysis, it was inferred that the H50Q mutant structure had a higher aggregation propensity than G51D and that these two mutations might have divergent effect on the fibrillation process of αS. Higher levels of contact area and intra-molecular residue-residue interactions were noticed in the case of H50Q when compared to the WT and G51D [251]. They observed the surface area, atomic contact energy (ACE) and geometric complementarity score to be the highest for H50Q and least for G51D. Then, they analyzed the typical intermolecular interactions that drive the association of two monomers of H50Q, WT and G51D. They noticed the interface residues and non-bonded contacts for H50Q (902) to be higher than WT (489) and G51D (333) protein. Therefore, they inferred from the interaction study that mutant H50Q can accelerate the aggregation propensity while G51D decelerates the aggregation propensity with respect to the WT αS. From the negative total binding free energy, it was observed that the homodimeric H50Q complex was more stable than the WT and G51D [251]. These results indicated that the individual structures of the WT and G51D mutant are thermodynamically more preferred than the individual structures of the H50Q αS. Therefore, it was noticed that the binding free energy holding the two monomeric units in H50Q dimer to be larger. Based on these calculations, it was predicted that the mutant H50Q readily forms a homo-dimer (early event of aggregation), whereas G51D and WT αS much less so. Using TANGO algorithm, the overall aggregation propensity of H50Q variant was shown to increase considerably when compared to those of the WT and G51D. This analysis helped in finding long-rang interactions that can promote protein aggregation due to the exposure of amino acid stretches around glutamine 50. Therefore, it was concluded that H50Q can aggregate at a much faster rate when compared to WT and G51D αS [251].

Tsigelny et al. generated different structural conformations of αS using implicit MD simulations and then examined secondary and tertiary structural changes in the conformers along the MD traces [252]. Then, they analyzed the membrane interaction of the conformers found along the MD traces. They elucidated the main regions of the proteins that interacted with the membrane and calculated percentages of these regions for WT and each mutant αS. The WT and mutant αS conformers were then used in attempts to estimate whether they would be able to form the propagating dimers and annular oligomers using the consecutive docking procedure to evaluate the possibility of quaternary structural formation [252]. As a result, the possible annular oligomers were obtained that were embedded to the membrane and then underwent MD equilibration in the membrane surrounded by explicit solvent to estimate the stability of the possible transmembrane channels created by WT αS and its mutants. Next, the possible protein–lipid contacts were analyzed for each ring structure and the intermolecular interactions between the neighboring αS monomers within the annular oligomers were studied [252]. The general scheme of three-level sets of protein residue interactions made it possible to suggest the points of drug interactions. Their study showed that increased oligomerization of the αS mutants might be associated with their greater membranephilicity and a propensity to penetrate the membrane. The snapshots of all mutant (A53T, A30P, E46K and H50Q) and WT αS molecules during MD were analyzed for their propensity to interact with the membrane. It was demonstrated the presence of a domain that showed increased membrane penetration [252].

The presence of defined domains within αS that interact with the membrane is consistent with the findings of previous studies that used fluorescent probes and ESR to show that the N-terminal region was the most immersed in the membranes around residue 3 (corresponding to Zone1), followed by the region around residue 90 (Zone4) and that the C-terminal region was the least immersed [253]. MD simulations were performed with the explicit solvent method for a maximum of 65 ns for the 1–99 fragment of αS. In all these cases, the highest level of protein motion occurred in two regions: around residues 39–45 (equivalent to Zone2) and around the residues 64–74 (equivalent to Zone3). Furthermore, Fantini and Yaji used monolayer experiments with short peptides derived from the αS sequence and MD simulations to demonstrate that tyrosine 39 insertion into the membrane is a molecular basis for the glycosphingolipid-binding specificity of αS and its membrane penetration [254]. The same sphingolipid-binding domain is also common for Aβ and prions [255].

Di Pasquale and coauthors, using MD simulations and electrophysiological measurements, showed that the major effects of the E46K mutation were the alteration of the channel properties of αS oligomers and the generation of nonstop activity [256]. This mutation is located directly in the 35–46 region (Zone2), which is a bend/loop break between the helices in the membrane-bound αS and is a main candidate for initiation of the membrane penetration of the protein. NMR studies showed that micelle-bound αS contains two α-helical regions: V3–V37 and K45–T92 with unstructured C-terminal tail. The unstructured loop including residues 38–44 (Zone2) links these two helices. A break in the helical structure at region 36–45 (Zone2) of the vesicle-bound αS has also been found using NMR. Helix breaks at residues 35–43 (Zone2) were demonstrated using electron spin resonance and MD simulations [27,257].

Evidence has accumulated regarding the secretion of αS in extracellular space and its interaction with specific lipids [256,258,259]. Using the in-cell NMR technique, it was demonstrated that αS preserves mostly disordered structure inside the cell [260,261]. Furthermore, according to the solution NMR analysis, in αS bound to the SDS micelles, the α-helical regions remained mostly stable [247]. Three regions underwent transformations into unstructured loops: 39–45 (Zone2), 63–67 (Zone3), and, to a lesser extent, 83–87 (Zone4). These results corresponded to the outputs of the MD simulations, where there were four main zones of contact of αS conformers with the membrane, with the Zone2 contacts having significantly greater propensity of membrane binding and penetration [252]. Finally, MD simulations results agreed with a study that used a combination of solid-state and solution NMR spectroscopy to characterize the conformations of αS bound to lipid membranes [262]. This study showed three αS regions, including an N-terminal helical segment performing the role of membrane anchor, an unstructured C-terminal region that is weakly associated with the membrane and a central region acting as a sensor of lipid properties and determining the affinity of αS membrane binding [262]. It was found that αS mutants have a greater propensity to interact with and penetrate the membrane through the domain of Zone2 in comparison with the other Zones, which is more frequent in the generated conformers with the best energies that in dimers and oligomers. These oligomers have significant interaction with the membrane, as they form possibly pore-like structures able to lead to outer ions influx and eventual cell death. These oligomers were stable in the membrane because of specific membrane–protein interactions: the oligomer rings interact with the hydrophilic groups in the top of the membrane that most probably organize the H-bonds with the lipid heads. In the middle hydrophobic area of the membrane, the ring proteins interacted via their hydrophobic residues, while, at the bottom of the membrane, proteins interacted using their hydrophilic residues. Such an arrangement leads to stable embedding of the rings in the membrane. Of the various mutations investigated by this group, their study showed that the E57K and A53T mutants adopted conformations that most frequently favored possible Zone2 interactions with the membrane. H50Q was the next in this frequency and A30P was the last.

The results of the MD simulation studies are in agreement with the results of western blot and immunogold analysis and are consistent with recent studies showing that E57K, followed by A53T and WT, has the most interaction power with the membrane [263]. The results were also in agreement with those produced by Ono and coauthors showing that A30P had less propensity to interact with the membrane [226]. Analysis of the frontal region of Zone2, the zone that would lead in membrane penetration, showed that, in all cases, K43 and/or K45 were present. They added additional mutations of these two residues to the oppositely charged aspartic acid and hydrophobic valine. These artificial mutations led to complete eradication of the Zone2 as a possible membrane-contacting region. Their predictions correlated with the experimental results demonstrating that the artificial K45E mutation of αS significantly delays its aggregation. The annular oligomers obtained from αS dimers have 5 to 7 monomers of αS. Interesting to note that a percentage of annular oligomers in relation to the total number of dimers for WT and mutant αS (one dimer can generate only one oligomer, some of the dimers can generate rings, some-fibrils and some do not generate any oligomeric structures) corresponds to experimental data. This percentage was higher for E57K, A53T and H50Q mutants and lower for E46K, E35K, WT and A30P mutants of αS that correlates with the experimental gold staining results. Further supporting these initial MD results was the fact that the tertiary structures changes reflected in the radii of gyration were consistent with the small-angle X-ray scattering (SAXS) results that demonstrated that the R_g_ for WT αS and its A30P, E45K and A53T mutants were between 20 and 42 Å [264]. In MD simulations for the WT αS and its mutants, the radii of gyration were in the range from 14 to 40 Å. Therefore, this study suggested that oligomer-prone αS mutants favor conformations that result in the increased interaction with the membrane.

The described simulation results helped in identification of the structural and/or thermodynamic properties of the WT αS protein and evaluated the impact of the artificial and/or pathological missense mutations on the structural and thermodynamic properties of WT αS at the atomic level with dynamics using MD simulations with or without special sampling techniques.

## 3. Understanding the Outputs of Artificial and Pathological Mutations in Aβ: Insights from the Molecular Dynamics Simulations

The formation of amyloid fibrils is a hallmark of many human diseases and results from the misfolding of proteins into cross β-sheet structure [82,265,266,267,268,269,270,271,272]. AD, for instance, is characterized by deposition of amyloid fibrils in the brain parenchyma and cortical blood vessels [273,274]. These deposits known as amyloid plaques consist of aggregates of 40- and 42-mer peptides (Aβ_40_ and Aβ_42_) produced through endoproteolysis of the precursor transmembrane protein by β- and γ-secretases [273]. Thus far, a high resolution structure for Aβ_40_ and Aβ_42_ is not available, in contrast to a seven-residue peptide fragment from a yeast protein Sup35 but we know that the β-strands run perpendicular to the fiber axis and the chains are in parallel register [275]. Several models have been presented based on solid-state NMR measurements, H/D exchange measurements with or without mutagenesis data and proline scanning methods [79,82,97,101,102,276,277,278,279,280,281,282,283]. These models have in common a disordered N-terminal region spanning at residues 1–10 and differ in the number and length of strands and loops and in the network of intermolecular hydrogen-bonding and sidechain-sidechain interactions.

The kinetic model, by which the Aβ peptides form the amyloid fibrils, is believed to follow a nucleation-growth model, with a lag phase of several days [89,150,284,285,286,287,288,289,290,291,292,293]. Oligomerization is very sensitive to amino-acid variations [123,294,295]. Aβ_42_ forms fibrils at a higher rate than Aβ_40_ and AD-causing A21G (Flemish) mutation (see above) has a slower aggregation kinetics than the WT Aβ and the E22Q (Dutch), E22K (Italian), E22G (Arctics), or D23N (Iowa) mutations [294,296,297,298,299]. In contrast to the late aggregates or protofibrils, which have been rather intensively characterized by experiments, the structures of monomers and oligomers formed at the early aggregation steps are poorly understood due to their transient and highly heterogeneous nature. Artificial missense mutations are utilized for understanding the structure-function relationships and aggregation mechanisms of monomeric and oligomeric Aβ. Pathological missense mutations impact the structures and thermodynamic properties and therewith the aggregation kinetics of Aβ. MD simulations with or without special sampling methods have been performed widely to gain insights into the impact of mutations on the structural and thermodynamic properties of Aβ in an aqueous solution environment at the atomic level with dynamics.

It seems likely that various factors including β-structure formation and intramolecular peptide interactions facilitate monomeric Aβ interactions and that these structural characteristics drive the formation of toxic oligomers and amyloid fibrils [300,301]. Regarding Aβ_42_, formation of a highly abundant α-helix has been reported in the central hydrophobic core region (L17–A21; CHC) and β-structure has been detected in the N-terminal and C-terminal regions, with the latter being more prominent in the WT Aβ_42_ peptide [84,212,302,303,304,305,306,307,308]. In addition, the abundant β-structure formation in the C-terminal region of the WT Aβ_42_ peptide has been linked to the aggregation mechanism [84,212,305,306]. Furthermore, the stabilization of the turn conformation at A21–A30 in the WT Aβ_42_ has been associated with the formation of salt bridges and hydrophobic interactions [84,212,305]. Interestingly, prominent β-structure is formed at R5 located in the N-terminal region of Aβ_42_ and this residue forms also various stable intramolecular interactions with other residues of the peptide located in the N- and C-terminal or mid-domain (L17–G29) regions [84,212].

**Figure 3:**
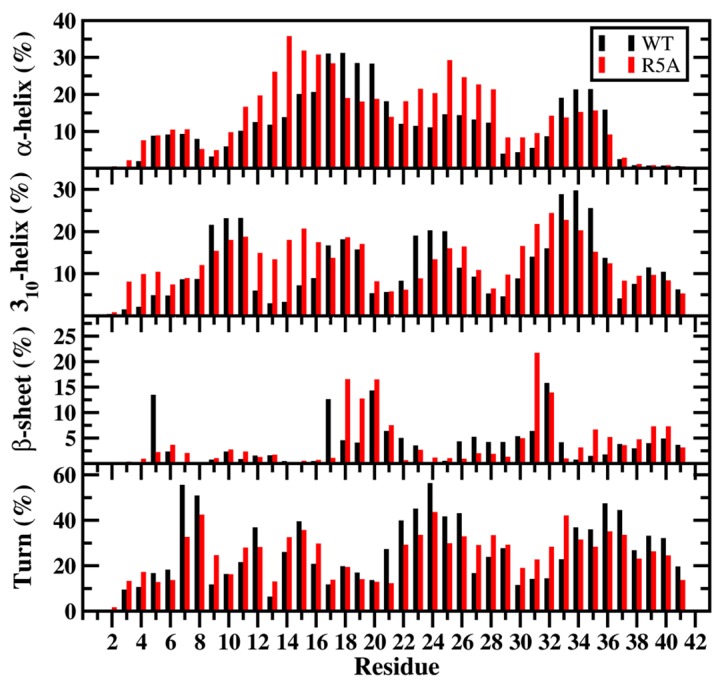
WT and R5A mutant Aβ_42_ secondary structure components. Secondary structures along with their abundances per residue for the WT (black) and R5A mutant (red) Aβ_42_ peptides per residue. The abundances for the π-helix and coil structures are not displayed.

Experimental R5A mutation studies showed a decrease both in the tendency toward Aβ aggregate formation and a reduced toxicity related to AD [309]. Further experimental studies showed that the R5A mutation depresses the interactions between Aβ and sphingomyelin, which also has been related to the degree of Aβ toxicity in AD [310]. In addition, blocking the E3–F4–R5–H6 region, in which the R5 residue is located, with targeted antibodies was shown to inhibit the Aβ aggregation [311,312,313]. These studies revealed that R5 stimulates the assembly of Aβ. However, the exact role of R5 and R5A mutation in the structures and free energy landscapes of Aβ_42_ in an aqueous medium at the atomic level with dynamics was not studied until Coskuner and Wise-Scira conducted REMD simulations to analyze the role of R5 in the structures of Aβ_42_ through R5A mutation [314]. They utilized the AMBER FF99SB parameters for the protein and the Onufriev–Bashford–Case implicit solvent model for water (see above). Each REMD simulation utilized 24 different replicas with temperatures exponentially distributed between 280 and 400 K. Both peptide structures were simulated for 300 ns per replica (for each peptide) with exchanges between replicas attempted every 5 ps, yielding a total simulation time of 7.2 μs and an exchange probability of 0.74 for both the WT and R5A mutant Aβ_42_ peptides. The calculated secondary structure components per residue showed that the β-sheet formation at L17, E22 and S26–G29 that occurs in the structures of the WT Aβ_42_ peptide almost completely disappeared upon R5A mutation in an aqueous environment (Figure 3) [314]. Residues V18–A21 located in the CHC region, as well as I31 and the C-terminal region except G33, G37 and I41 adopted more abundant β-sheet structure upon R5A mutation. A significant difference in the N-terminal region was noticed at E3–H6 and V12–K16, since these segments adopted more abundant 3_10_-helix upon R5A mutation of the Aβ_42_ peptide. Furthermore, more prominent α-helical structure formation occurred at Y10–K16 and E22–I32 regions of the peptide as a result of the R5A mutation [314]. Less abundant α-helix and 3_10_-helical structure were formed at residues Y10–V12, L17–A21, A21–D23 and G33–V36 in R5A mutant. The impact of R5A mutation on the structural stability of the Aβ_42_ peptide in an aqueous environment was studied using both harmonic and quasi-harmonic methods (Table 2). A comparison of the thermodynamic values revealed that the R5A mutation increased the conformational enthalpy (*H*) value by ~840 kJ·mol^−1^. Although a drastic change in the conformational entropy value (−*TS*) upon R5A mutation was not observed utilizing the harmonic method, the conformational entropy values calculated using the quasi-harmonic method showed a decrease by 370 kJ·mol^−1^ upon R5A mutation (Table 2). Nevertheless, the R5A mutation resulted in less stable structures of Aβ_42_ regardless of the chosen thermodynamic method. These results suggested that R5A mutation destabilizes the structures of Aβ_42_ in an aqueous environment [314]. The β-sheet structure almost disappeared in the Ala21–Ala30 region but was more abundant in parts of the central hydrophobic core and C-terminal regions of Aβ_42_ upon R5A mutation. More abundant α-helix was adopted in parts of the N-terminal and mid-domain regions and less prominent α-helix formation occurred in the central hydrophobic core region of Aβ_42_ upon R5A mutation. Interestingly, intramolecular interactions between N- and C-terminal or mid-domain regions disappeared upon R5A mutation [314]. The structures of Aβ_42_ were thermodynamically less stable and showed reduced compactness upon R5A mutation. R5A mutant structural stability increased, with more prominent central hydrophobic core and mid-domain or C-terminal region interactions. Based on these results, small organic molecules and antibodies that inhibit the β-sheet formation in the Ala21–Ala30 region and hinder the intramolecular interactions occurring between the N-terminal and mid-domain or C-terminal regions of Aβ_42_ may help to reduce Aβ_42_ toxicity in AD [314].

The 10th amino acid in the primary structure of Aβ_42_ is Tyr, which is reported to be active toward zinc, copper and iron binding and also plays central roles in nitration and phosphorylation of Aβ_42_ [153,154,155,315,316,317,318,319]. Furthermore, interactions with ligands and receptors are dominated by Tyr and are proposed to be neurotoxic or neuroprotective since they impact the aggregation rate and kinetics of Aβ_42_ [153,154,155,315,316,317,318,319]. Dysfunction of intrinsically disordered Aβ_42_ due to misfolding and failure to fold into the appropriate conformation needed for certain functions lead to the increased toxicity and aggregation. The precise molecular mechanisms of AD pathogenesis remain poorly understood. Various factors including point mutations, binding of transition metals and the absence or presence of post-translational modifications have been proposed. Early characteristics associated with the pathological developments are changes in cellular levels of Aβ, misfolding of this peptide and metabolic dysfunction.

Coskuner and Murray investigated the association between the metabolic dysfunction and the structures of the Aβ conformational ensemble [320]. Specifically, they analyzed whether adenosine triphosphate (ATP) alters misfolding of Aβ_42_ and reported a link between ATP binding and Aβ structure. The Tyr10 residue in the primary structures of Aβ_42_ was found to interact significantly with ATP [320]. The ability of these interactions to cause misfolding was shown theoretically and validated by experiments. Specifically, biochemical experiments showed that ATP reduced Aβ misfolding at physiological intracellular concentrations with the threshold values at 500 μM and 1 mM. Tyr interactions with ATP were shown to be specific and became stronger in the presence of magnesium [320]. Tyr-gated electron transfer was shown to play a central role in the toxicity of Aβ [321]. Nevertheless, the specific roles of Tyr in the Aβ_42_ structure remained to be investigated. Both neuroprotective and neurotoxic effects have been reported for Tyr-bound ligands in the structures of Aβ. Understanding of the impact of the aromatic Tyr residue and the Tyr10Ala mutation on the monomeric conformational ensemble of the Aβ_42_ peptide was crucially needed.

**Figure 4:**
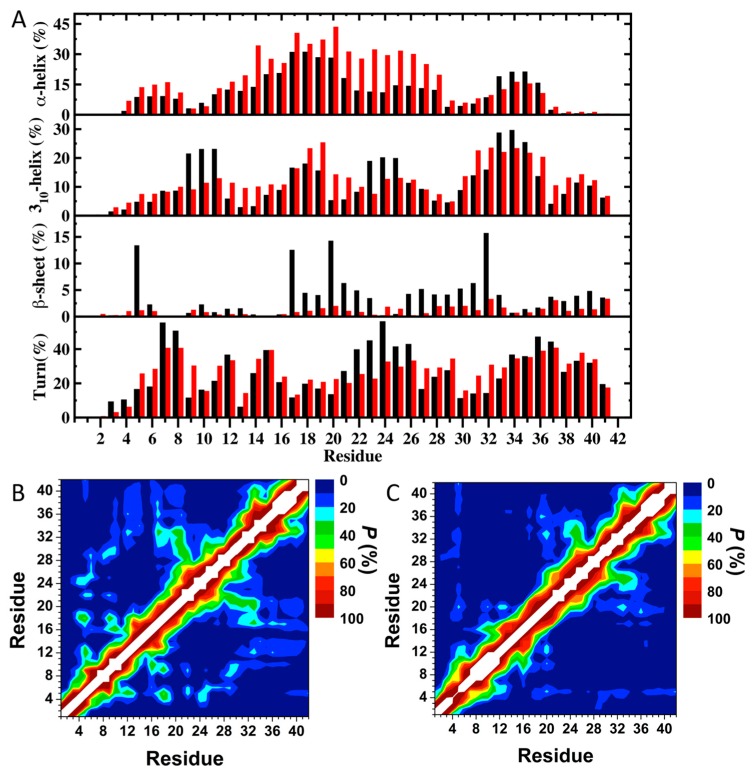
(**A**) Secondary structure components per residue along with their abundances per residue for WT Aβ_42_ (black) and the Tyr10Ala mutant (red) in an aqueous solution. The abundances of the π-helix and coil conformations are not displayed; (**B**) Tertiary intramolecular interactions and abundance of tertiary contacts in the structure of WT Aβ_42_; (**C**) Tertiary intramolecular interactions and abundance of tertiary contacts in the structure of the Tyr10Ala mutant. In last two plots, the color scale corresponds to the probability (P) that the distance between the centers of mass of two residues is ≤9 Å.

**Figure 5:**
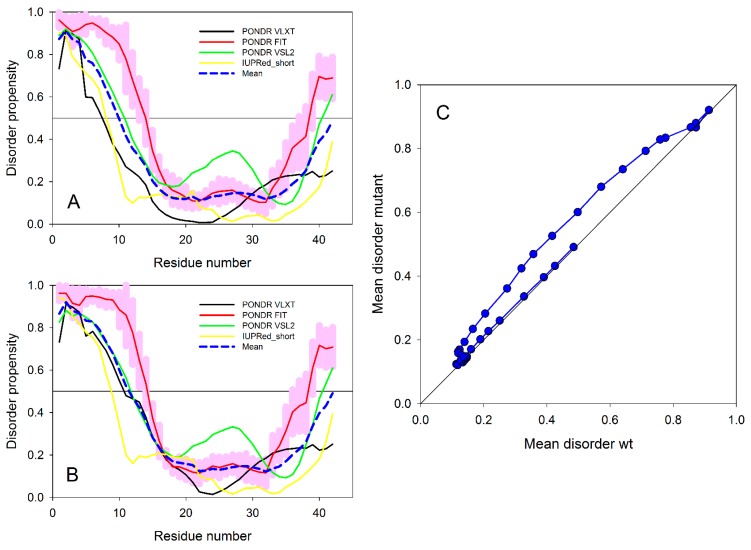
Analysis of the effects of the Tyr10Ala mutation on the intrinsic disorder propensity of human Aβ_42_ was evaluated using several common disorder predictors, including PONDR FIT, PONDR VLXT, PONDR VSL2 and IUPred. Scores above 0.5 are considered to correspond to disordered residues/regions. (**A**,**B**) Results of this multiparametric computational analysis of intrinsic disorder predisposition for (**A**) WT Aβ_42_ and (**B**) the Tyr10Ala mutant; (**C**) Per-residue mean disorder propensity calculated for the Tyr10Ala mutant vs. the per-residue mean disorder propensity calculated for WT Aβ_42_.

**Figure 6:**
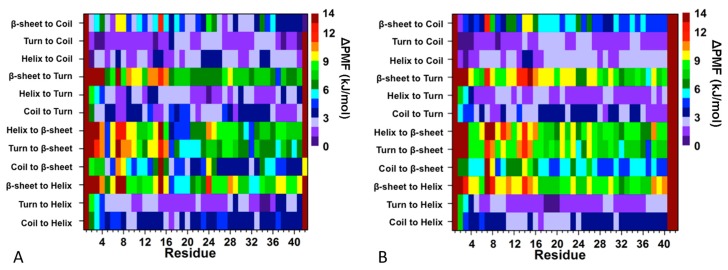
Secondary structure transitions between two specific secondary structure components per residue for (**A**) WT Aβ_42_ and (**B**) the Tyr10Ala mutant in an aqueous solution medium. The color scale corresponds to the free energy values associated with specific transitions between two secondary structure components for a specific residue.

Coskuner and Uversky investigated the role of Tyr in the structures of Aβ_42_ through Tyr10Ala mutation using sets of REMD simulations with different force field parameters [322]. Various sets of REMD simulations of the WT Aβ_42_ and Tyr10Ala disordered peptides in an aqueous environment were conducted. The AMBER ff14SB and CHARMM22/CMAP force field parameters were utilized for the disordered peptides in separate simulations. The Onufriev-Bashford-Case implicit model for water and the modified TIP5P model for water were used for modeling the solvent (see above) in separate simulations. The secondary structure and thermodynamic properties were investigated using harmonic and quasi-harmonic methods. Overall, the results of this study showed that the structural and thermodynamic properties of WT Aβ_42_ in an aqueous medium were significantly altered as a result of the Tyr10Ala mutation. The analysis showed that the β-structure formation in Aβ_42_ is regulated by Tyr, which promoted β-sheet ordering in the disordered structures of the Aβ_42_ conformational ensemble (Figure 4A). On the other hand, the Tyr10Ala mutation strengthened the disordered nature of Aβ_42_ in terms of conformational enthalpy, entropy (harmonic and quasi-harmonic) and Gibbs free energy, as well as secondary and tertiary structure properties (Table 3, Figure 4) and intrinsic disorder propensity as evaluated by a set of common disorder predictors (Figure 5). The Tyr10Ala mutation yielded less compact structures in comparison with the WT Aβ_42_ (cf. Figure 4B,C). Surprisingly, the Tyr10Ala mutation caused a significantly larger decrease in the overall abundance of β-sheet formation or led to the disappearance of β-structure in members of the Aβ_42_ conformational ensemble [322]. In view of the crucial role of β-structure formation in the reactivity of Aβ_42_ toward ligand and receptor interactions, including Aβ_42_ self-oligomerization and fibrillation (see above), these findings suggested that the Tyr10Ala mutation decreased the reactivity of Aβ_42_ toward various ligands and self-oligomerization in aqueous environments [322]. These findings were supported by evaluation of the aggregation predisposition by a set of computational tools developed to find aggregation-prone regions in a query protein. Overall, these observations were in accord with the experiments conducted by Tu and Raleigh, Terol et al. and Bemporad et al., who studied the impact of Tyr mutation on various proteins other than WT Aβ_42_ [323,324,325]. These authors detected slower aggregation kinetics caused by Tyr mutations. Since β-sheet structure formation represents the lead mechanism in self-assembly, these results showed that Tyr10Ala mutation reduces the β-structure formation in Aβ_42_, which in turn may slow down the oligomerization, fibrillation and aggregation kinetics. The secondary structure transition stabilities (obtained by the TISS method) reveal that the formation of coil structure rather than β-structural or α-helical conformations is preferred upon Tyr10Ala mutation (Figure 6) [322].

Vu and co-authors investigated the structural change of the 3 Aβ_11–40_ upon F19W mutation over 400 ns in temperature REMD simulations with 48 replicas [326]. They calculated the free energy values using the MM/PBSA method (see above). Their results showed that the secondary structure terms slightly change upon F19W mutation, since mutant showed 3% less β-sheet and 3% more coil contents. In addition, the number of contacts, SASA and radius of gyration values changed only slightly upon F19W mutation. However, RMSD values showed an increase caused by the F19W mutation. Important polar contacts between D23 and residues 24–29, which help stabilizing the loop region, showed a decrease by 20% upon mutation. The similarity in structural terms and higher binding affinity between constituting chains indicated that the hydrophobic core of Aβ was capable of adapting flexible changes. However, the decrease in critical polar contacts, higher fluctuation in binding energy, higher number of minima with lower energy barriers and significantly lower population indicated a significant increase in the flexibility of the mutant. These results also contributed to the understanding of the fibrillation of Aβ. The more flexible F19W mutant Aβ oligomers would require a larger time to self-assemble into fibrils, which is consistent with the REMD simulation results that the mutation results in longer lag phase [326].

The amyloid channel hypothesis that postulates the presence of pore structures formed by small oligomers that are capable to disrupt cellular ionic homeostasis is emerging as one of the principal hypotheses associated with pathogenesis of protein deposition diseases [118,327,328,329,330,331,332]. Of particular interest is the point mutations clustered around a central hydrophobic region of Aβ. These include the E22Q point mutation, associated with hereditary cerebral hemorrhage by amyloidosis of the Dutch type; the E22G mutation (see above) and the A21G mutation (see above), related to cerebral amyloid angiopathy and presenile dementia [333,334]. Proline mutations in this central region have attracted particular interest, as they have been shown to suppress β-sheet and fibril formation in the Aβ peptide and fragments thereof [283,335,336,337]. The β-strand conformation in individual Aβ peptides has been modeled as being essential for the formation of cell membrane-penetrating pores [338,339,340]. Cysteine mutations have also been investigated in this central region, with L17C and V18C point mutations resulting in a decreased level of fibril formation and with F20C producing a degree of fibril formation similar to that of WT Aβ_40_ [278,281].

**Figure 7:**
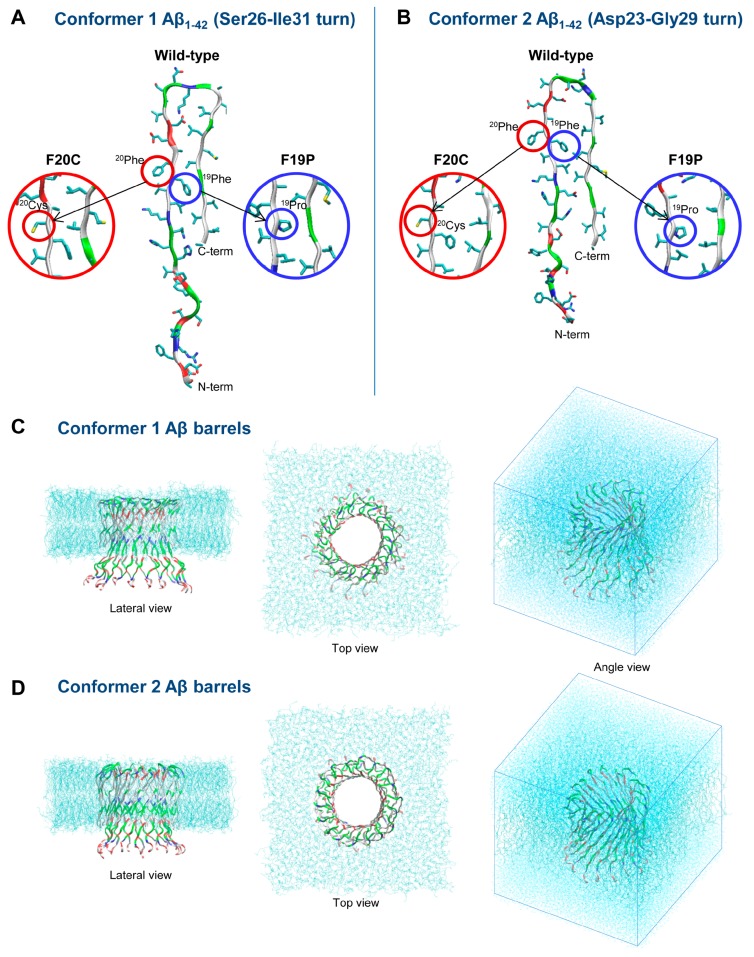
Monomer conformations of the Aβ_42_ wild type and F19P and F20C mutants with different turns at (**A**) Ser26–Ile31 (conformer 1) and (**B**) Asp23–Gly29 (conformer 2). Starting points of the Aβ_42_ barrels embedded in the lipid bilyer for the MD simulations for (**C**) conformer 1 and (**D**) conformer 2 Aβ_42_ barrels. Waters were removed for the sake of clarity in the lateral and top views but they are depicted as cyan dots in the simulation box in the angle view. In the peptide ribbon, hydrophobic residues are colored white, polar and Gly residues are green, positively charged residues blue and negatively charged residues red.

Nussinov and co-authors used MD simulations to investigate the effect of F19P and F20C mutations on the pore structures formed by the full-length Aβ_42_ peptide inside the lipid bilayers [341,342,343,344]. The anionic lipid bilayer containing a total of 420 lipid molecules constituted the unit cell with the TIP3P water molecules, added at both sides of the bilayer. The CHARMM27 force field parameters were used for proteins. The propensity of the F19P mutant to form channels was found to be similar to that of the wild type peptide through AFM imaging in a DOPC bilayer [341]. MD simulations also predicted channel formation, however, with a collapsed or clogged pore for the two available solid-state NMR-based Aβ_42_ conformers (see Figure 7). This is in agreement with the electrophysiology studies, which reported no ionic conductance by the F19P mutant [341]. The proline substitution is known as a β-structure breaker. This indicated a role for the β-sheet in the Aβ pore and argued for further studies of the effect of this mutation on peptide conformation during channel formation. The degree to which the β-sheet was disrupted by this mutation is still unclear and is likely to vary in the heterogeneous channel landscape. Because of the compromised structure and activity of the F19 in ion conductance and the β-sheet structure stabilization, it may be a viable target for the AD therapeutic development against pore conductance. Structurally, the F20C mutant was found to behave similar to the wild type peptide both in MD simulations and in AFM imaging of pore formation [341].

The sequence of Aβ_25–35_ (GSNKGAIIGLM) has a positively charged N-terminus and a hydrophobic C-terminus. The solution structures of the Aβ_25–35_ are a mixture of random coil, β-strand and α-helix [345,346]. Hydrogen/deuterium (H/D)-exchange NMR experiments indicate that the Aβ_25–35_ amyloid fibrils have a core formed from residues 28–35, with residues 31 and 32 being the most protected from H/D exchange [347]. Even though the H/D-exchange NMR results indicate that N27 is only marginally protected in the Aβ_25–35_ amyloid fibril, the Aβ_25–35_ Asn27Gln mutant does not form amyloids [348]. It seems that the difference in amyloid formation for the Aβ_25–35_ and Asn27Gln mutant does not come from the perturbation of the amyloid fibril core. To understand why the N27Q mutation blocks in vitro amyloid formation, Nussinov and co-workers carried out exhaustive simulation studies of both Aβ_25–35_ and Asn27Gln mutant sequences to investigate (1) the stability of the candidate amyloid oligomers and (2) the distributions of free energies for candidate intermediate monomer states with partial secondary structure formation [349]. These MD simulations did not show destabilization effects of the Asn27Gln mutation of the oligomer clusters of Aβ_25–35_. In contrast, the relative conformational stabilities of the Aβ_25–35_ monomers were altered in the Asn27Gln mutant, which may slow the amyloid formation process. The structure and stabilities of the partially folded intermediates affect protein folding as well as misfolding and amyloid formation. By applying Kramer’s theory of barrier crossing and a Morse-function-like energy landscape, it was shown that the intermediates with medium stability dramatically increased the rate of amyloid formation [349]; on the other hand, very stable and very unstable intermediates sharply decreased amyloid formation [350]. Remarkably, extensive molecular dynamics simulations and conformational energy landscape analysis of the Aβ_25–35_ and its N27Q mutant corroborated the mathematical description. Both experimental and simulation results indicated that the core of the amyloid structure of Aβ_25–35_ is formed by the residues 28–35. A single mutation of N27Q of Aβ_25–35_ made the Aβ_25–35_ N27Q mutant amyloid-free [349]. Energy landscape calculations showed that the Aβ_25–35_ peptide conformational ensemble included the extended intermediates with medium stability that were prone to form amyloids, whereas the extended intermediates of the Aβ_25–35_ N27Q mutant were split into stable and very unstable species that did not show the predisposition to form amyloids. The results of this study explained the contribution of both α-helical and β-strand intermediates to amyloid formation [349].

Substitution of aspartic acid at the position 23 with tyrosine is known to drastically accelerate amyloid formation by Aβ peptide. The importance of the position 23 also follows from the observations that its mutation promotes fibril growth [351]. Thus, by performing REMD simulations, Takeda and Klimov sought to provide a microscopic explanation for these findings [352]. Furthermore, studying the fibril growth for the mutant Aβ offered a direct computational test to the hypothesis that side chain interactions may impede fibril growth. This aspect of their study bears some general interest in the context of the role of sequence in amyloid formation. The system included four peptides forming a fibril fragment and two incoming peptides interacting with the fibril. 24 replicas were distributed linearly in the temperature range from 330 to 560 K with the increment of 10 K. They produced seven REMD trajectories resulting in a cumulative simulation time of 34 μs. Using REMD simulations they probed the effect of Asp23Tyr mutation on the mechanism of Aβ_10–40_ fibril growth. The consequences of the mutation were evaluated by computing binding free energy landscapes, distributions of peptide-fibril interactions and through the comparison with the WT Aβ_10–40_ peptide. They showed that Asp23Tyr mutation had limited impact on the docking of Aβ peptides to the fibril, which remained barrier-less. In contrast, the locking stage was strongly affected by the mutation due to the profound stabilization of the parallel in-registry β-strands formed by the peptides on the fibril edge. The enhanced stability of the parallel β-sheets resulted from the deletion of strong side chain interactions formed by Asp23, which were incompatible with the locked state. Based on the simulation data, it was expected that Asp23Tyr mutation would promote fibril growth. The analysis of Asp23Tyr mutation therefore suggested that strong off-registry side chain interactions may slow down fibril assembly as it occurs for the WT Aβ peptide. This observation can be useful in predicting the effects of mutations on fibril growth. The available experimental data appear to support their in silico conclusions [352].

The three GxxxG motifs spanning between residues 25 and 37 have a major impact on the Aβ_42_ peptide aggregation and membrane perturbation, processes that have been implicated in oligomer toxicity [353,354,355]. Biophysical studies showed that the G25L, G29L, G33L and G37L mutants of Aβ_42_ undergo β-sheet and fibril formation at an increased rate compared with WT Aβ_42_. The accelerated rate of amyloid fibril formation resulted in a reduced population of dimeric and trimeric forms of Aβ in solution, as detected by mass spectrometry [354]. On the basis of in vitro and in vivo experiments, Harmerier et al. found that Aβ_42_ oligomers with substitution of glycine 33 by alanine and isoleucine were much less toxic than the WT Aβ_42_ peptide, suggesting that G33 may represent the critical residue linking toxicity and oligomerization [353]. Using different techniques, it was also shown that both Aβ_42_ G33A and G33I mutants promoted the aggregation process in vitro by increasing the population of large oligomers at the expense of small oligomers. However, how these mutations affected structures of the early Aβ_42_ oligomers was not established. Derreumaux and co-workers applied REMD simulations with the OPEP coarse grained protein force field to the Aβ_29–42_ fragment for understanding the impact of both G33A and G33I substitutions on the earliest steps along the Aβ_42_ aggregation pathway [356]. They utilized an implicit model for water. The percentage of β-strand, coil, turn and bend as calculated by the DSSP program amounted to 44%, 9%, 7% and 38% for WT, 24%, 20%, 11% and 36% for G33A and 32%, 22%, 14% and 27% for G33I. The CD spectrum of Aβ_29–42_ at 22 μM showed a maximum β-sheet content of 50% within a few hours. We know, however, by solid-state NMR that Aβ_34–42_ forms amyloid fibrils with antiparallel β-sheets [357]. These simulations on the dimers at 292 K pointed to a β-strand content of 57%, 65% and 37% for WT, G33A and G33I, respectively and a high population of antiparallel β-strands with various registers for the three alloforms [356]. They also revealed that an increase of hydrophobicity at G33 enhanced the population of parallel orientations, albeit with a low probability (<11%). The calculated side-chain–side-chain contact probability map for WT Aβ_29–42_ dimer displayed an interpeptide antiparallel organization for the L34–A42 segment, consistent with the solid-state NMR data for Aβ_34–42_ fibrils. It was noted that the conformational ensemble for the WT Aβ_29–42_ dimer was partially consistent with the simulations performed by Itoh and Okamoto, in which a much higher population of α-helical structures was observed [358]. The balance between intra- and intermolecular interactions determines the oligomerization properties; i.e. the rate of fibril formation and the populations of oligomers of different sizes. OPEP-REMD simulations on the monomer showed that the β-strand content at 297 K varies in the order WT > G33I > G33A [356]. While the WT peptide had a significant propensity for β-hairpin (69%), consistent quantitatively with the previous all-atom REMD simulations in solution (53%) and qualitatively with the computational study of Itoh and Okamoto, the G33A peptide had a much lower probability (21%) and even more striking was finding that the G33I peptide had a very moderate probability to adopt β-hairpin structures (7%) [356,358]. Interestingly, the OPEP-REMD dimer simulations provided another β-strand order, G33A > WT > G33I and showed that dimerization proceeded through different interactions. The assembly of Aβ_29–42_ G33I and G33A dimers was driven by the main-chain and side-chain interactions between the N-terminal residues 31–35, whereas in the WT Aβ_29–42_ dimer, the intermolecular interactions between the C-terminal residues 35–41 were the dominant [356]. Comparing the three alloforms, this study revealed that the G33I monomer had the highest population of coil-turn structures (94%) and the G33I dimer displayed a higher conformational freedom of the C-terminal residues with reduced intermolecular and long-range intramolecular interactions than the G33A dimer and then the WT dimer. These results strongly indicate that the population of the amyloid-competent conformations followed the order G33I > G33A >WT in Aβ_29–42_, providing, therefore, on the basis of another computational study, a plausible explanation for the faster aggregation rates and shorter lag phase times of the two Aβ_42_ G33A and G33I mutants. Another finding of biophysical studies on Aβ_42_ peptides was that the substitution of G33 by alanine, isoleucine or leucine significantly decreased the population of small oligomers, namely dimers and trimers, for G33L and G37L6 and from dimer to dodecamer for G33I. Though it remains to be determined whether the results on Aβ_29–42_ can be extrapolated to Aβ_42_ (the role of the N-terminal (1–10) and hydrophobic (16–21) residues is well documented) these simulations showed that the presence of a long hydrophobic stretch spanning residues 30–36 followed by another hydrophobic stretch spanning 39–42 disrupts many intermolecular contacts and makes it very difficult to protect all the hydrophobic side chains from the solvent, reducing therefore the lifetimes of the dimers of Aβ_29–42_ G33I and G33A. They hypothesized that only higher-order assemblies allow the burial of the hydrophobic surface in Aβ_42_ G33I, G33A and G33L variants, since there is computational evidence that hydrophobic interactions drive the early aggregation steps of Aβ_42_ [356].

Derreumaux and co-workers studied the impact of A2V mutation on the structures of dimeric Aβ_40_ using REMD simulations [359]. All simulations were carried out with 60 replicas varying from 300 to 448 K. Each replica was run for 400 ns. The A2V mutation was reported to protect from AD in its heterozygous form and cause an early AD type dementia in its homozygous form [105]. Experiments showed that the aggregation rate followed the order A2V > WT > A2V-WT [105]. To understand the impact of this mutation, REMD simulations of Aβ_1–40_ WT-A2V and A2V-A2V dimers was carried out and compared to the WT dimer [359]. Their atomistic simulations revealed that the mean secondary structure remained constant but there were substantial differences in the intramolecular and intermolecular conformations upon single and double A2V mutations. Upon single mutation, the intrinsic disorder was reduced, the intermolecular potential energies were reduced, the population of intramolecular three-stranded β-sheets was increased and the number of all α dimer topologies was decreased [359]. Taken together, these results offered an explanation for the reduced aggregation rate of the Aβ_1–40_ A2V-WT peptides and the protective effect of A2V in heterozygotes.

It has been reported that an A2T mutation in Aβ can protect against AD. Interestingly, a nonpolar A2V mutation also has been found to offer protection against AD in the heterozygous state, although it causes early onset AD in homozygous carriers [360]. Since the conformational landscape of the Aβ monomer is known to directly contribute to the early-stage aggregation mechanism, it is important to characterize the effects of the A2T and A2V mutations on Aβ_42_ monomer structure. Belfort and co-workers compared the monomeric conformational ensembles of the A2V and A2T Aβ_42_ variants with that of WT by performing extensive atomistic REMD simulations in explicit water [361]. First, a 10 ns MD simulation at high temperature (~700 K) in vacuum was performed starting from a fully extended peptide conformation with charged termini. The collapsed peptide was solvated in a 56 × 56 × 56 Å^3^ cubic box containing ~5600 water molecules. The solvated peptide was equilibrated for 2 ns in an NPT ensemble (300 K and 0.1 MPa) before the REMD run. Finally, constant volume REMD simulations were run for 175 ns per replica with an integration step of 2 ns, resulting in an aggregate simulation time of 11.2 μs per system. A total of 64 replicas within an exponentially distributed temperature range of 276–592 K were used for each system and the replica exchange attempts were made every 4 ps. Taken together, their findings suggested that the effect of the second amino acid on the Aβ_42_ monomer structure is highly complex and sequence-dependent. An enhanced double-hairpin population similar to those reported in toxic WT Aβ_42_ oligomers was found in the A2V monomer [361]. Hydrophobic clustering between the N-terminus and the central and C-terminus hydrophobic patches promotes such double-hairpin formation in A2V. In contrast, the A2T mutation triggered unusual ionic interactions of the N-terminus with K16 and E22, thereby impeding CHC-CTR hairpin formation. Consequently, a unique population comprising only the C-terminal hairpin was observed. Although further investigation is needed to obtain a complete molecular picture of the relationship between monomer misfolding, aggregation and toxicity and protection against or causation of AD by these N-terminal variants, the simulations described therein clearly show that single A2V and A2T substitutions can alter the structural landscape of the Aβ_42_ monomer by shifting the equilibrium to different conformational states [361].

Derreumaux and co-workers took the NMR solid-state structure of Aβ_40_ fibril and constructed the initial structures for Aβ_42_ and its A21G variant (see above) using the SWISS-MODEL server [362]. All Aβ models were solvated in a rectangular 90 × 50 × 40 Å box with 6000 simple point-charge water molecules and simulated using periodic boundary conditions. The particle-mesh Ewald method was used with a cutoff distance of 12 Å. MD simulations were performed using the NPT ensemble. All Aβ models were simulated for 10 ns at 400 K to increase phase space sampling. Overall, they studied the impact of the point mutation A21G on the structure of Aβ dimers, by using a total of six unfolding MD simulations at 400 K. They obtained a time-averaged β-percentage of 26% (Aβ_42_, Aβ_40_-A21G), 35% (Aβ_40_) and 40% (Aβ_42_-A21G) excluding the first 5-ns and considering residues 1–8 disordered. Aβ dimers were found in equilibrium between a wide range of topologies, ranging from four-stranded to seven-stranded β-sheets, with the strands S2 being very mobile and the location of the strands S1 fluctuating between residues 11–20 (in Aβ_40_) and residues 13–16 (in Aβ_40_-A21G).

This finding raises the question whether a unique inhibitor can block propagation of these structurally distinct dimers into protofibrils. Secondly, the effect of A21G mutation on Aβ dimers is length-dependent and the structures and dynamics of Aβ_42_-A21G cannot be extrapolated from those of Aβ_40_-A21G and vice versa [362]. This is consistent with earlier experimental studies suggesting that substitutions at positions 22 and 23 produce different effects on Aβ assembly depending on whether they occur in Aβ_40_ or Aβ_42_ [89]. Specifically, it was found that the A21G mutation impacts Aβ dimers in three ways: A21G destabilizes the β-sheets and notably strands S2 in Aβ_40_ but not in Aβ_42_; A21G also increases, to a higher extent, the flexibility of the central hydrophobic cluster spanning residues 17–21 in Aβ_40_ than in Aβ_42_; and affects, to various degrees, the populations of the intramolecular and intermolecular salt bridges involving Glu22, Asp23 and Lys28 in Aβ_40_ and Aβ_42_ [362]. These three factors likely slow down the formation of higher-order species to direct further assembly into protofibril and could explain the reduced aggregation rate of Aβ fibrils containing the Flemish disease-causing mutation.

Urbanc and co-workers studied the effects of the arctic (E22G) mutation on Aβ folding using discrete MD (DMD) simulations [363]. Specifically, they examined folding of full-length Aβ_40_ and Aβ_42_ and their Arctic mutants, using DMD combined with a four-bead protein model and implicit solvent interactions. The temperature-induced conformational transitions obtained in silico were consistent with in vitro experiments that showed conformational transitions from a collapsed coil at low temperatures to β-strand-rich extended conformations at higher temperatures [364]. Consistent with the CD measurements by Lim et al. they observed a faster increase in the average amount of β-strand in Aβ_42_ relative to Aβ_40_ [365]. Their model predicted the central folding region centered at G25–S26 in both Aβ_40_ and Aβ_42_ and the C-terminal folded structure centered at G37–G38 in only Aβ_42_, in agreement with the in vitro findings of several groups. Existing experimental and all-atom MD studies on the fragment Aβ_(10–35)_ are consistent with their observation of the collapsed coil monomer structure dominated by loops, bends and turns at low temperatures [366,367,368,369]. Their results demonstrated that small changes in the primary structure can have significant impact on folding, suggesting that full-length Aβ_40_ and Aβ_42_ and their mutants need to be examined to gain insights into pathological differences between the alloforms. Their study extends our understanding of how the additional amino acids I41 and A42 at the CTR of Aβ_42_ significantly impact full-length Aβ folding. The more hydrophobic CTR of Aβ_42_ is known to facilitate structural changes resulting in different oligomerization pathways and pathologies of Aβ_40_ and Aβ_42_. Bitan et al. reported that Aβ_40_ forms smaller oligomers (from dimers to tetramers) while Aβ_42_ forms larger oligomers (pentamers/hexamers) and their multiples [370].

Urbanc and co-workers demonstrated that structural differences between Aβ_40_ and Aβ_42_ that mediate this distinct oligomerization behavior already exist in the isolated monomers [363]. The structural difference between the two alloforms at the C-terminus, a turn centered at G37–G38 in Aβ_42_ but not in Aβ_40_, seems to be a direct consequence of two additional hydrophobic amino acids at the C-terminus of Aβ_42_. However, the folding differences between Aβ_40_ and Aβ_42_ at the N-termini, the β-strand at A2–F4 in Aβ_40_ but not in Aβ_42_ as well as a β-hairpin centered at S8–Y10 in Aβ_42_ but not in Aβ_40_, were surprising. This structural difference at the N-terminus of Aβ_40_ versus Aβ_42_ has not been reported experimentally, to our knowledge. Hou et al. studied Aβ with reduced and oxidized M35 and showed that a turn or bend-like structure at D7–E11 in oxidized peptides was less frequent than in the reduced peptides [371]. Examining folding of the two Arctic mutants, [G22]Aβ_40_ and [G22]Aβ_42_, they showed that the presence of Gly22 disrupts contacts close to position 22 and importantly, also at the N-terminus of Aβ_40_, resulting in a [G22]Aβ_40_ conformer that is structurally similar to Aβ_42_ in this region [363]. The average amount of β-strand formed at a physiological temperature in [G22]Aβ_40_ was higher than in [G22]Aβ_42_. Their observation that the substitution E22G increases the propensity for β-strand formation is not surprising. This substitution not only reduces the overall negative charge of the Arctic peptides but also, through the G22 substituent, increases the local backbone flexibility needed for a collective hydrogen bond ordering into a β-strand. The Arctic mutation did not significantly alter the structure of Aβ_42_. Instead, the major effect appeared to be on the secondary structure of Aβ_40_, which was more “Aβ_42_-like.” The increased level of regular secondary structure in Aβ_40_ is likely to affect its oligomerization pathway, as observed in vitro and in vivo. Several studies have reported that the Arctic mutation significantly increases the protofibril formation rate relative to the wild type. Their simulation result for [G22]Aβ_40_ showed an increase in the average β-strand propensity when compared to the wild type, which was consistent with experimental findings [363].

Dahlgren et al. developed two aggregation protocols for the production of stable oligomeric or fibrillar preparations of Aβ_42_ and its Dutch (E22ΔQ) and Arctic mutants [371]. In terms of neurotoxicity, the wild type and the mutants were not significantly different but extensive protofibril and fibril formation by the mutant peptides was observed [90]. Experimental studies by Murakami et al. demonstrated that the mutations at positions 22 and 23 played a significant role in self-assembly of Aβ peptide [114]. Specifically, the Arctic mutant showed a 50% increase in the average β-strand content in Aβ oligomers. Whalen et al. found that Arctic Aβ had an increased rate of assembly into oligomers and that these oligomers were more toxic to neurons in culture than were wild type oligomers [372]. These experimental findings on Arctic peptides are consistent (from simulations) with the increased β-strand propensity in folded Arctic monomers relative to their wild type counterparts. Take together with other data extant, Urbanc and co-workers suggested that small changes in the primary structure of Aβ not only may affect peptide monomer folding itself but also changes the rate of formation, structure and neurotoxic properties of higher order assemblies [363].

Shea and co-workers studied the effects of single amino acid substitutions associated with the Italian (E22K), Arctic (E22G), Dutch (E22Q) and Iowa (D23N) familial forms of AD and cerebral amyloid angiopathy on the structure of the 21–30 fragment of Aβ using REMD simulations [373]. The OPLS-AA (proteins) and TIP3P model for water were used in these simulations [373]. The 21–30 segment had been shown in earlier work to adopt a bend structure in solution that may serve as the folding nucleation site for Aβ [374,375,376,377,378]. Their simulations reveal that the 24–28 bend motif is retained in all E22 mutants, suggesting that mutations involving residue E22 may not affect the structure of the folding nucleation site of Aβ [373]. Enhanced aggregation in Aβ with familial AD substitutions may result from the depletion of the E22–K28 salt bridge that destabilizes the bend structure. Alternately, the E22 mutations may affect longer-range interactions outside the 21–30 segment that can impact the aggregation of Aβ. Substituting at residue D23, on the other hand, leads to the formation of a turn rather than a bend motif, implying that in contrast to E22 mutants, the D23N mutant may affect monomer Aβ folding and subsequent aggregation. Their simulations suggested that the mechanisms by which E22 and D23 mutations affect the folding and aggregation of Aβ were fundamentally different [373].

Sticht and co-workers conducted MD simulations to study the impacts of E22G, E22K, E22Q and E22Δ on the structures of Aβ [379]. All simulations were conducted using the AMBER FF99SB parameters for the proteins and TIP3P model for water. An unconstrained 20 ns production phase with standard NPT conditions at 1 bar was performed for data collection. The free energies were calculated using the MM/GBSA method (see above). The mutant oligomers themselves exhibit a higher internal stability due to the lack of electrostatic repulsion between adjacent E22 residues. Secondly, the mutant oligomers can no longer be incorporated into fibrils. Together, both effects suggest a mechanism for the enrichment and enhanced stability of Aβ-oligomers observed in experimental studies. Dual effect of E22 mutations offers an explanation, why changes at this sequence position have such a drastic effect on the progression of the disease. Finally, their findings affirm the oligomer hypothesis of AD that claims prefibrillar aggregates and oligomers to be direct effectors of synaptic and cognitive dysfunction in AD [379].

In a recent study, intact lipid bilayers were exposed to predominantly monomeric preparations of WT or different mutant forms of Aβ_40_ and atomic force microscopy (AFM) was used to monitor aggregate formation and morphology as well as bilayer integrity over a 12 h period [380]. The goal of this study was to determine how point mutations in Aβ, which alter peptide charge and hydrophobic character, influence interactions between Aβ and the lipid surface. The Arctic, Italian, Iowa and Flemish mutations (see above) were considered. While fibril morphology did not appear to be significantly altered when mutants were prepared similarly and incubated under free solution conditions, aggregation in the lipid membranes resulted in a variety of polymorphic aggregates in a mutation dependent manner. It was further found that the ability of Aβ to disrupt the structural integrity of bilayers was notably modulated by these mutations. An enhanced bilayer disruption was reported for the Arctic mutation. It was speculated that, in comparison to WT Aβ, the increased hydrophobic nature of E22G Aβ increases its bilayer insertion. The membrane-bound oligomers of the Iowa mutation were extremely stable and the bilayer developed small, discrete areas of disrupted lipid morphology. Based on the overall electrostatic and hydrophobic properties of D23N Aβ this finding could not be explained.

The goal of the MD simulation study performed by Strodel and co-authors was to investigate the effects of the charged residues K16, E22, D23 and K28 on the stability of transmembrane Aβ_42_ in a zwitterionic palmitoyl-oleoyl phosphatidylcholine (POPC) bilayer and their role on membrane integrity [381]. Their 500 ns MD simulations of Aβ_42_ mutants in a POPC bilayer reveal a similar or increased stability compared to WT Aβ_42_ for all mutants except D23G. For the monomeric β-sheet they observed the highest stability for the Arctic mutant E22G and the double mutant K16M/K28M. The removal of positive charges by mutating K16 and K28 to methionine increased the hydropathy index of this mutant Aβ_42_ by a factor of 2.34, which gave rise to a stable transmembrane β-sheet. The stability of the Arctic mutant E22G can be attributed to the removal of the negative E22 charge in combination with D23 and K28 interacting with the head groups of the lower leaflet, leading to charge neutrality of the peptide inside the membrane. While the ‘Arctic-type’ D23G mutant has the same hydropathy index as E22G Aβ_42_, it is not stable as transmembrane β-sheet, since the position of E22 inside the membrane causes the peptide to bend towards the upper membrane surface. The less toxic WT Aβ_42_, on the other hand, loses some of its β structure during the MD simulation due to its overall negative charge inside the membrane [381]. For APP, it was experimentally shown that the Arctic mutation altered the transmembrane localization compared to WT APP, leading to the reduced levels of Arctic APP at the cell surface making it less available for non-amyloidogenic cleavage. As a result, the extent and subcellular location of Aβ formation was changed, as revealed by increased Aβ levels, especially at intracellular locations. Their simulation results revealed that also for Aβ the Arctic mutation increases its propensity to remain buried inside the lipid bilayer [381]. NMR studies have revealed a large destabilizing effect of the D23G mutation on the turn region involving residues 21–30, which is in agreement to their computational results of transmembrane D23G Aβ_42_ [382]. Experimental studies of Aβ mutant peptides revealed that the aggregation propensity to form (proto)fibrils is not sufficient to explain the observed in vivo toxicity of the Aβ_42_ peptides [115,383]. These simulation results on the interactions between Aβ_42_ and a POPC bilayer and the effect of Aβ_42_ mutations on bilayer properties provided further insight into the likely toxicity mechanism caused by membrane-inserted Aβ_42_ oligomers. It was concluded that the higher transmembrane stability of E22G and its increased membrane disturbing effect, compared to WT Aβ, were possible reasons for the increased cytotoxicity of Arctic Aβ. While their current simulations were still rather short investigating only small oligomers (simulations of larger than tetrameric oligomers on the millisecond time scale would be needed, which are yet prohibitively long with respect to computing time) they were able to state that the Aβ_42_ mutations have an effect on transmembrane stability and membrane integrity [381]. This should be motivation enough for experimentalists and simulation scientists to perform further studies on these systems.

Proteolysis experiments on Aβ_40_ and Aβ_42_, under conditions favoring oligomerization, identified a protease-resistant segment, Ala21–Ala30, in both peptides [377]. The homologous decapeptide Aβ_21–30_ shows identical protease resistance [377]. Structure calculations based on the distance constraints from proton solution-state NMR of Aβ_21–30_ revealed a turn structure in the Val24–Lys28 region [377]. Lazo et al. postulated that this structure nucleates the intramolecular folding of the Aβ monomer and that partial unfolding of the Ala21–Ala30 region may be necessary for the subsequent fibrillation of Aβ [377]. The observations on full-length Aβ and the Aβ_21–30_ decapeptide are consistent with previous work showing that peptide fragments containing the folding nuclei of globular proteins are, by themselves, structured [384]. Furthermore, the structures found in the isolated folding nuclei were similar to those found in the full-length proteins. MD simulations of the folding nucleus, therefore, provide insights into the earliest events in the folding of the full-length protein.

Borreguero et al. recently used discrete MD with implicit solvent and a united-atom protein model to simulate folding of the putative Aβ_21–30_ folding nucleus [378]. The united-atom peptide model considers explicitly all protein atoms except hydrogen. Important findings in Borreguero et al. are: (i) the existence of a loop that is stabilized by hydrophobic interactions in the Val24–Lys28 region; (ii) a high degree of flexibility in the termini; and (iii) electrostatic interactions between the charged groups of Glu22, Asp23 and Lys28 that modulate the stability of the folded structure [378]. Stanley and co-workers tested whether the stability of the Val24–Lys28 loop described by Borreguero et al. persists in simulations that consider an explicit solvent (all atoms are included in the simulation) [376]. In addition, they determined the effects of solvent alterations on the folding dynamics and investigated the changes in the dynamics caused by amino acid substitutions. They also studied the dynamics of the monomer containing the Dutch [Gln-22]Aβ_21–30_ mutation. They used the CHARMM27 force field parameters for the proteins and the TIP3P model for water. They solvated each monomer by inserting it in the center of a previously equilibrated cube of water molecules of side 43 Å. This insertion deleted all water molecules overlapping or in close proximity (>2.4 Å) to any of the monomer atoms, resulting in a system with 2542 water molecules. They generated five trajectories with the following initial conditions: [RC], WT random coil conformation in normal density water; [P1] and [P2], WT loop conformations from Lazo et al. in water with reduced density, [DU], Dutch peptide in a random coil conformation in normal density water; and [RCS], WT random coil conformation in salted water (see above). Total simulation times for the production runs were 102.6 ns for [RC], 65 ns for [P1], 83.6 ns for [P2], 80.0 ns for [DU] and 145.0 ns for [RCS]. Their simulation results showed that hydrophobic interactions play a crucial role in Aβ_21–30_ folding dynamics, assisted by the formation of salt bridges between the charged amino acids. By performing secondary structure and hydrogen bond analysis, they found that there is no regular secondary structure or permanent hydrogen bonding, suggesting that folding involves formation of a loop stabilized by the packing of the side chains of Val24 and Lys28. They also show that by reducing the density of water they may induce formation of a π-helix [374]. Interestingly, they found that in normal density water, if the solvent contains desolvated ions, the salt bridges play a prominent role in the stabilization of the Val-24–Lys-28 loop. Finally, they reported that for the Dutch [Gln22]Aβ_21–30_ decapeptide in water, elimination of charge at position 22 disrupts the natural tendency of the monomer to form a long-lived Val24–Lys28 loop. This substitution likely alters the Aβ folding pathway, leading to the formation of alternative turn structures, including those stabilized solely by an Asp23–Lys28 salt bridge [376].

Pande and co-workers conducted Markov state model analysis to sample an exceptional submillisecond timescale (greater than 200 μs) using an explicit model for water and characterized the structures of full-length Aβ_40_ and Aβ_42_ monomers [385]. In addition to the effects of peptide length, they reported the impact of Italian mutation E22K (see above) on Aβ structures [385]. Aβ_42_, Aβ_40_ and Aβ_42_-E22K monomers were largely unstructured, with a slight tendency to form short α- and 3_10_-helix segments. The helix-forming tendency was strongest in the region of residues 10–20 for Aβ_42_ and Aβ_40_. Some formation of β-hairpins was also observed, mostly employing a glycine in their turns. β-Hairpins between residues 30, 31 and residues 34, 35 (using I32 and G33 as its turn) and between residues 35, 36 and residues 39, 40 (using G37 and G38 as its turn) are most populated in Aβ_42_. The formation of both these β-hairpins is substantially decreased in Aβ_40_ [385]. The pathogenic Aβ_42_-E22K forms β-hairpins to a similar extent and at the same locations as the WT Aβ_42_ but notably has increased α-helix formation in the region of residues 20–24. These induced helix fragments in the region of residues 20–24 may increase helix-helix interactions between Aβ_42_-E22K monomers and could lead to alignment of unstructured regions nearby the helices, thus promoting oligomerization [385].

**Figure 8:**
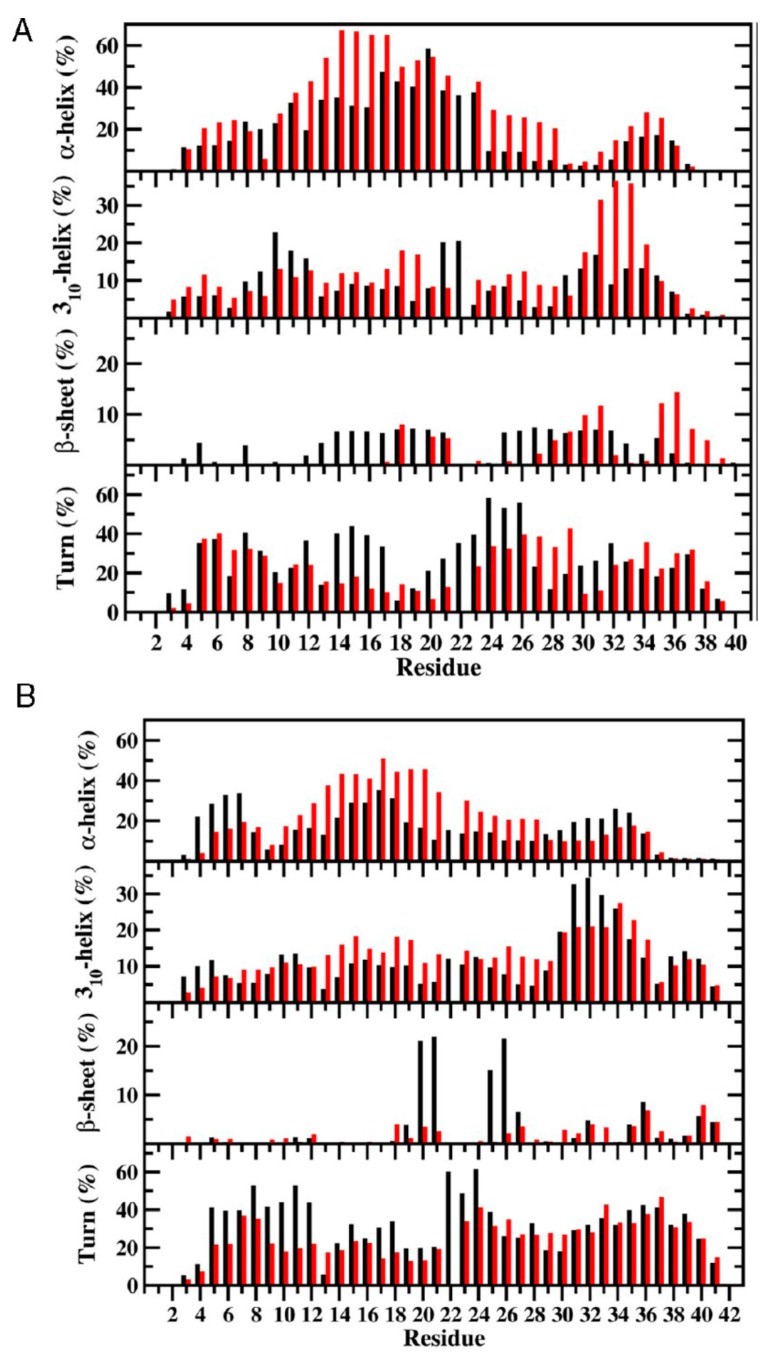
Calculated secondary structure abundances per residue for the (**A**) WT (black) and E22Δ mutant (red) Aβ_40_ and (**B**) WT (black) and E22Δ mutant (red) Aβ_42_ peptides in aqueous solution. The abundances for the π-helix and coil structures are not displayed.

Kitahara and co-workers studied the impact of the Osaka (E22Δ) mutation on the structures of Aβ_40_ and Aβ_42_ in an aqueous solution environment using REMD simulations [212]. The REMD simulations were performed utilizing the Amber ff99SB potential function and Onufriev–Bashford–Case generalized Born implicit solvent model for the protein and solution environment, respectively (see above). An implicit solvent model was chosen to avoid configurational sampling limitations due to confined aqueous volume effects [188] and inaccuracies in the specific heat of constant volume REMD simulations with explicit water reported by Parrinello and co-workers [386]. The temperatures of each replica for each peptide were exponentially distributed between 280 and 400 K, yielding exchange ratios of approximately 0.74 for all four Aβ peptides. Each system was simulated for 100 ns for each replica (for each peptide) with a total simulation time of 2.4 μs. They calculated the conformational free energy values for all peptides using the MM/PBSA method (see above) [212]. The most abundant residual secondary structures were the coil and turn conformations in all wild- and mutant peptides (Figure 8). The average overall abundances of α-helix in the N-terminal region (Asp1–Lys16) of the WT Aβ_40_ and Aβ_42_ and the E22Δ mutant Aβ_42_ peptides were similar (17% and 19%). However, the overall α-helical structure abundance was larger (30%) in the same region of the E22Δ mutant Aβ_40_, indicating that E22Δ mutation increases α-helix prominence in the N-terminal region of Aβ_40_ (Figure 8). Specifically, Arg5–Asp7 and Tyr10–Lys16 in Aβ_40_ showed an increase upon E22Δ mutation (up to 36%) [212]. Using specific residual analysis, they detected that Phe4–Asp7 of Aβ_42_ adopted less abundant α-helix (≤19%) upon E22Δ mutation while an increase in α-helix prominence (up to 21%) was observed for Ser8–Lys16 (Figure 8). The overall β-sheet contents in the N-terminal regions of the wild- and E22Δ mutant Aβ_42_ were small (0.4–0.9%). The WT Aβ_40_ presents slightly larger overall β-sheet content in the N-terminal region (2.3%) which disappears upon E22Δ mutation (Figure 8), indicating that the oligomerization and fibrillation process involving the β-sheet forming residues located in the N-terminal region of Aβ_40_ was less likely in the E22Δ mutant Aβ_40_ peptide in comparison to its WT form (Figure 8). Regarding the formation of the turn structure differences, the largest discrepancies occurred at His14–Lys16 of Aβ_40_ (with a decrease up to 29%) and at Arg5–Val12 of Aβ42 (with a decrease up to 35%) upon E22Δ mutation (Figure 8). The overall α-helix abundance was 9.5% and 12.7% larger in the mid-domain region (Leu17–Ala30) of the E22Δ mutant Aβ_40_ and Aβ_42_ in comparison to the WT Aβ_40_ and Aβ_42_, respectively (Figure 8). Specifically, Leu17–Phe19, Ala21 and Asp23–Lys28 adopted more prominent α-helix (up to 9%) in the structures of E22Δ mutant Aβ_40_ in comparison to the WT Aβ_40_ peptide (Figure 8). The same residues and additionally Phe20 formed more prominent α-helix (up to 28%) in the structures of Aβ_42_ upon E22Δ mutation (Figure 8). The same region presented a slight decrease in the β-sheet prominence in the structures of the E22Δ mutant Aβ_40_ (2.0%) and Aβ_42_ (4.9%) in comparison to their WT forms (Figure 8). Namely, Leu17, Phe19–Ala21 and Gly25–Lys28 adopted less prominent β-sheet in the structures of Aβ_40_ and Aβ_42_ upon E22Δ mutation. Interestingly, the overall turn structure abundance decreased by 6.4% and 7.3% in the structures of E22Δ mutant Aβ_40_ and Aβ_42_ in comparison to the WT Aβ_40_ and Aβ_42_ peptides, respectively [212]. The turn structure formation in the Ala21–Ala30 region has been related to the distinct structuring of the disordered protein as well as to its aggregation mechanism and toxicity. Kitahara and co-workers found that Ala21–Ser26 and Ala30 of E22Δ mutant Aβ_40_ and Ala21–Gly25 and Lys28 of E22Δ mutant Aβ42 adopted less abundant turn structure by 12–34% and up to 58% in comparison to their WT forms, respectively. These results might indicate that Ala21–Ala30 is less reactive toward aggregation in the structures of the E22Δ mutant Aβ_40_ and Aβ_42_ peptides in comparison to the same region in the WT Aβ_40_ and Aβ_42_ peptides. β-Structure in the central hydrophobic core (CHC; Leu17–Ala21) region decreased its abundance to 7% and 18% in the structures of Aβ_40_ and Aβ_42_ upon E22Δ mutation, respectively. This finding indicates that the reactivity of the CHC region toward aggregation is depressed in E22Δ mutant in comparison to the WT Aβ alloforms. For the C-terminal region, they noted an interesting trend in the helix structures; Ile31–Met35 adopted more abundant α-helix in the structures of the E22Δ mutant Aβ_40_ in comparison to the same region in its WT form, while the opposite trend was detected for the same residues of the E22Δ mutant and WT Aβ_42_ peptides (Figure 8). Furthermore, 3_10_-helix formation increased sharply for Ile31–Leu34 in the structures of the E22Δ mutant Aβ_40_ peptide in comparison to its WT form (Figure 8). On the other hand, 3_10_-helix formation was decreased by 11–14% at Ile31–Gly33 in Aβ_42_ upon E22Δ mutation (Figure 8). The β-sheet formation in the C-terminal region of the E22Δ mutant Aβ_42_ did not differ significantly from that of the WT Aβ_42_ that they reported most recently. However, Ile31, Met35–Val39 adopted more abundant β-sheet (up to 12%) in the structures of the E22Δ mutant Aβ_40_ peptide in comparison to its WT form (Figure 8) [212]. This result indicates an increased tendency toward aggregation via the C-terminal region of the E22Δ mutant Aβ_40_ in comparison to the WT Aβ_40_ peptide. Overall, the calculated secondary structure properties for the WT alloforms, for which experimental and theoretical data exist, are in excellent agreement with previously performed studies [84,305,306,308,365].

The calculated conformational Gibbs free energies using harmonic and quasi-harmonic methods are listed along with the enthalpy and entropy values in Table 4. The same trends were observed using either a harmonic or a quasi-harmonic method in simulations. Namely, the structures of the WT Aβ_42_ were less stable than those of the WT Aβ_40_ peptide in an aqueous solution environment [212]. These conformational Gibbs free energies supported previous studies that reported a higher tendency toward aggregation for the WT Aβ_42_ rather than the WT Aβ_40_ (see [212] and references therein). Both E22Δ mutant alloforms were less stable than their WT forms but the trend between the two different alloforms was inversed upon E22Δ mutation. Specifically, the E22Δ mutant Aβ_42_ structures were more stable than those of the E22Δ mutant Aβ_40_. Based on these findings, the E22Δ mutant Aβ_40_ has a slightly larger tendency toward aggregation in comparison to the E22Δ mutant Aβ_42_ peptide in an aqueous solution environment [212].

Nussinov and co-workers performed explicit MD simulations of octadecameric (18-mer) Osaka mutant (ΔE22) and wild type (WT) Aβ_42_ barrels in a DOPC bilayer [387]. The monomer mutant conformation was derived from the Aβ_42_ peptide via a deletion of Glu22. Two U-shaped mutant conformers, with a turn at Ser25–Ile30 for conformer 1 and a turn at Asp22–Gly28 for conformer 2, inherited the same turn conformations from the Aβ_42_ conformers with slightly different turns, Ser26–Ile31 in conformer 1 and Asp23–Gly29 in conformer 2. However, the pore-lining residues inverted their side-chain orientation due to the deletion of the residue. They showed that the ΔE22 mutant formed a β-barrel-like channel in the lipid bilayer, with the membrane embedded conformation indistinguishable from the WT Aβ_42_ barrel. For the conformer 1 ΔE22 barrel, they obtained outer and pore diameters of ~7.91 and ~2.1 nm, respectively, indicating that the overall dimensions were consistent with the Aβ_42_ barrels in their study. Further, these dimensions were also in the range of the outer diameter, ~7.8–8.3 nm and pore diameter, ~1.8–2.2 nm, for the same 18-mer Aβ_42_ barrels in the anionic bilayer composed of DOPS/POPE. However, for the conformer 2 ΔE22 barrel, they obtained outer and pore diameters of ~7.62 and ~1.52 nm, respectively, with the overall dimensions being slightly reduced as compared to the Aβ_42_ barrels. The decrease in the pore diameter resulted from the inverted Lys16 side-chains that unfavorably reside in the central hydrophobic core between the β-sheets (z = ~0.5 nm). As a result, the unfavorable force exerted on the backbones of pore-lining residues slightly relocates them toward the pore axis. This caused reduction in the pore diameter and hence in the outer diameter of the barrel as well. In contrast, the conformer 1 of ΔE22 barrel could preserve the overall dimensions consistent with the WT barrels, since the Lys16 side-chains are located very close to the amphipathic interface of the lipid bilayer at the lower bilayer leaflet (z = ~−1.0 nm), stretching to interact with the C-termini or phosphate groups of lipids. The heterogeneity in the dimension of ΔE22 barrels suggested that ΔE22 may be less toxic than WT Aβ_42_, since the mutant barrels with smaller pores would be populated. Recently, it was shown that ΔE22 exhibits less neurotoxicity than WT Aβ_42_ in rat primary neurons. It is interesting to note the implications for WT Aβ from the Osaka mutant barrels. The coordinates for conformer 1 Aβ_42_ were directly extracted from the NMR-based Aβ_42_ fibrils (PDB code: 2BEG). For the conformer 2 Aβ_42_, they obtained the coordinates from the Aβ_40_ protofibrils (PDB codes: 2LMN and 2LMO) and then added two C-terminal residues, Ile41 and Ala42, generating Aβ_42_. Although both conformers are the same U-shaped peptide with the β-strand-turn-β-strand motif, they can be distinguished by their different turns. This suggests that the turn at Asp23–Gly29 of the conformer 2 should belong to an intrinsic turn of Aβ_40_. A similar turn at Val24–Ala30 for Aβ_40_ was recently identified from a structural model of Aβ_40_ fibrils also using comprehensive solid-state NMR techniques [387]. Thus, the conformer 2 Aβ_42_ adopting the Aβ_40_ turn topology is likely to be a relatively less populated conformation than the conformer 1 Aβ_42_, suggesting an explanation for the solid state NMR observation for this turn for the Aβ_40_ peptide rather than the conformation of the Aβ_42_ [100]. If the WT Aβ_40_ were to adopt the turn conformation of Aβ_42_, then the C-terminal strand will be too short to create a stable U-shaped conformation. Therefore, while the less C-terminal turn conformation of Aβ_42_ was more stable, the resulting shorter strand for the 1–40 sequence shifted the equilibrium toward the more C-terminal turn conformation.

Recently, it was demonstrated that the C-terminal domain of Aβ_42_ showed a distinct conformational dynamics from that of Aβ_40_, which suggested that the Val36–Cly37 turn was the *sine qua non* of Aβ_42_ [388]. For the Osaka mutant, the conformer with the less C-terminal turn was still more populated, since the shortened sequence with the Glu22 deletion affected the N-terminal strand, rather than the C-terminal one, thus retaining an equilibrium favoring the Aβ_42_ turn. The negatively charged Glu22 was implicated as a key site for point mutation, since amino acid substitutions at position 22 include Dutch (E22Q), Arctic (E22G) and Italian (E22K) mutants [116,299,388,389,390]. While the Dutch mutation is closely associated with cerebral amyloid angiopathy [391], other mutants with a familial AD (FAD) linked point substitution at Glu22 are toxic species, suggesting that Glu22 plays a significant role in the AD pathogenesis [115]. However, in spite of the complete elimination of Glu22, rather than an amino acid substitution, Aβ peptide lacking Glu22 is still linked to FAD. Previously, it was evaluated that in the solvated pore, the charged side-chain of Glu22 plays an important role in conducting ions in the amyloid ion channel models [115,338,342,344]. This evaluation was supported by the observation that a circular cluster formed by the negatively charged Glu22 side-chains attracted cations into the pore and served as a cationic binding site [338,342,344]. The larger the ions population at the binding site, the higher the probability for ions to conduct through the water pore. However, in the absence of Glu22, the ΔE22 barrels still attracted cations into the pore and showed large charge fluctuations in the pore similar to the WT barrels. Although the ΔE22 barrels lose the negatively charged Glu22 side-chains at the upper bilayer leaflet, they gain the Glu11 side-chains at the lower bilayer leaflet [387]. Thus, the emerging Glu11 side-chains attract cations into the pore and contribute to pore elongating with the β-sheet formation. To evaluate the biological role of a charged side-chain in the pore, it was suggested that a circular cluster of charged side-chains can be formed with a help of ion screening. This minimizes the charge repulsions between the ions, with the circular assembly of side-chains strongly sustaining the backbone β-sheet formed by the pore-lining residues. With well-established pore, which is wide enough, ions can move freely through the water pore. Even though the Osaka mutant that they have modeled also appears to adopt a toxic channel conformation consisting of a barrel organization of the U-shape motif, this does not necessarily imply that such conformational species are always the preferred conformational states. As they have already emphasized the amyloid landscape is highly heterogeneous and different conformations may be populated, including in membrane-permeated channels, suggesting that highly polymorphic conformations of Aβ channel could evolve from different seed formations. It is a challenge to infer all possible highly populated states for different mutants, under different conditions and membrane compositions. Their MD simulations provide a membrane-bound conformation of the Osaka mutant barrel in atomic-level detail, illustrating that the multimeric β-barrel-like channel can be indistinguishable from the WT Aβ_42_ barrel [387]. The U-shaped peptide with the β-strand turn-β-strand motif supports the Osaka mutant barrel, suggesting the universality of the Aβ motif in aggregation. It has been known that due to the loss of charge, the mutant has higher hydrophobicity resulting in faster oligomerization and fibril formation [392,393,394]. They speculate that high production of mutant oligomers can lead to toxic channel formation followed by oligomers insertion into the cell membrane in FAD [387]. The mutant conformational dynamics along with the membrane insertion and channel formation have not been well elucidated. The conformational space of Aβ monomer, oligomer and membrane-embedded channel states is highly polymorphic, with the mutant sharing these free energy landscapes, however with varied conformational preferences. Of particular interest, this deletion mutant suggests an explanation for why the solid-state NMR data for Aβ_40_ presented a more C-terminal turn conformation versus Aβ_42_ and argues that the more toxic Aβ_42_ species would preferentially populate the less C-terminal turn. Those two extra residues at the C-terminus shift the free energy landscape toward the more stable conformation described by Lührs et al. [100]. Aβ_42_ lacking Glu22 still retain these conformational preferences, since the deletion affects the conformation of the N-terminal, rather than the C-terminal strand [387].

As aforementioned, it is well established that the E22Q peptide shows enhanced activity (as measured by the rate of deposition or fibril elongation) relative to the WT peptide for both the Aβ_42_-peptide and the Aβ_(10–35)_-peptide congener. For the Aβ_(10–35)_-peptide congener, the E22Q mutant form of the peptide was found to deposit at a rate 215% faster than the WT peptide [395]. In a study of Austen and coworkers, it was found that Aβ_40_-E22Q-peptide formed oligomers and fibrils more rapidly than the WT peptide [396]. Using CD spectroscopy, they observed that the rate of change from mainly random coil to β-sheet was more than one order of magnitude higher in the E22Q mutant than in the WT. It was also determined that the rates of conversion from random coil to β-sheet in the WT and E22Q mutant peptides, derived from CD measurements, were an order of magnitude lower than the rate of formation of low-molecular-mass oligomers. It was suggested that the Aβ-peptide aggregates in an irregular structure and then undergoes a slower conformational transition into larger aggregates of β-sheets. It has been proposed that the E22Q peptide has a propensity for the formation of β-structure in solution.

A study by Selkoe and co-workers explored the binding of heparin to solutions of WT and E22Q mutant Aβ-peptide [397]. Heparin binds to fibrillar but not to non-fibrillar, Aβ-peptide. It was found that the E22Q mutant peptide assumed conformations to which heparin would bind more readily than did the WT peptide and that the affinity of heparin binding to the E22Q mutant peptide was similar to the affinity for binding of heparin to preformed β-fibrils. The results led to the conclusion that the water-aggregated E22Q mutant peptide adopted structures similar to those found in certain β-fibrils. Through CD and FTIR measurements, Miravalle et al. found that whereas the WT and the E22K mutant peptide were largely in the random-coil conformation in solution, the E22Q peptide assumed a β-sheet conformation [116]. The study explored the time dependence of peptide aggregation by CD and showed that for their sample preparations, the WT, E22K and E22Q peptide converted to β-structure over a period of hours. Whereas CD spectra of the WT and E22K mutant peptide samples indicated that at the earliest times the peptide was in a random-coil conformation, the E22Q peptide sample showed clear signs of β-structure. However, the results in the case of the E22Q mutant peptide could be due to the presence of peptide aggregates from the earliest stages of the CD measurements.

This brief survey of recent experimental results raises two fundamental questions. In the monomeric Aβ-peptide, is there local “flickering” of conformations consistent with the larger scale formation of β-structure? Does the E22Q mutation lead to a greater propensity for the formation of β-structure in the monomeric Aβ peptide? MD simulations of the fully solvated WT Aβ_(10–35)_-NH2 peptide and the E22Q mutant peptide were performed by Straub and co-authors [398]. For the simulations of the WT and mutant peptides, the solute was centered in a rhombic dodecahedron cell that was carved from a cubic box of 50 Å on a side and then filled with 2113 water molecules. They used the CHARMM22 force field parameters for the proteins. After the equilibration period of 200 ps, a production run of 1 ns was completed with an average temperature of 300 K. Their simulation data indicated that the central core structures of the Aβ_(10–35)_-peptide, characterized by an LVFFA (17–21) hydrophobic cluster and VGSN (24–27) turn region, were stable in aqueous solution in both the WT and E22Q mutant sequences as observed in the NMR studies of Lee and coworker. The E22Q peptide was more flexible in solution, supporting an early hypothesis that the equilibrium structural fluctuations of the E22Q mutant peptide were larger than those of the WT peptide [398]. The peptide adopted predominantly helical-like structures and showed much weaker β-strand propensity in all trajectories. Stable helix was typically formed near the hydrophobic cluster region LVFFA (17–21). The peptide’s C terminus also displayed strong helical propensity. There was no consistent pattern of β-strand flickering in the WT or E22Q mutant peptide. These observations were consistent with studies by Teplow and colleagues who argue, based on CD spectra, that the formation of cross β-fibril structures may be preceded by helix formation [89]. Therefore, the transition from the random coil to fiber involve the pathway RC↔Helix↔β. Few direct side-chain interactions were observed between the LVFFA (17–21) and VGSN (24–27) regions in the WT peptide. However, in both the WT and E22Q mutant peptides it was observed that the LVFFA cluster and VGSN turn region interacted through hydrogen bonding. These simulation results did not support the hypothesis that the Dutch E22Q mutation leads to a higher probability of formation of β-structure in the monomeric peptide in aqueous solution. A number of experimental studies have concluded that the Dutch mutant of the monomeric Aβ-peptide undergoes structural transition to a β form in aqueous solution [116,396]. Their simulation results suggested that it is likely that those observations result from the formation of solvated oligomeric peptide clusters, where the peptide’s β structure is stabilized by the peptide–peptide interactions [398]. However, the simulation time in these experiments was extremely short (1 ns) and one cannot rule out the possibility that E22Q may exhibit a significant propensity for forming β-structures on timescales much longer than 1 nanosecond.

Shea and co-workers studied the same E22Q mutation (see above) using MD simulations [399]. They used OPLS-AA force field parameters for the proteins and the TIP3P water model for the solvent. Three sets of simulations were performed. First, structural characterization of the Aβ_15–28_ (sequence: QKLVFFAEDVGSNK) and its E22Q mutant were carried out. To improve conformational sampling, the constant-pressure REMD algorithm was used. A total of 60 replicas of the original system were considered, at temperatures exponentially spaced between 277 and 600 K. Both Aβ_15–28_WT and E22Q mutant were acetylated and amidated at the amino and carboxyl terminus, respectively and solvated in a cubic box with length of 4.5 nm containing 3000 water molecules. Two trajectories were generated, 25 ns each. Refolding of a soluble conformation of Aβ into a conformation found in amyloid fibrils is the first and critical step on the pathway that leads to fibril growth [399].

In the past few years, a number of hypotheses have been put forth to explain how the E22Q mutation affects the refolding process of Aβ in an attempt to rationalize the effect of this mutation on the rate of fibril formation [116,398,399,400]. These hypotheses involve (a) a proposed reduction in the β-helical structure in the 10–24 N-terminal domain of Aβ accompanying the E22Q mutation; (b) an overall increase in β-sheet structure in the E22Q Aβ mutant; and (c) unfolding of the CHC upon E22Q mutation. Hypotheses (a) and (b) are not particularly compelling because previous FTIR and more recent NMR studies show little difference in overall secondary structure between the WT and mutated peptide and no dramatic conformational change upon mutation. The third hypothesis [399] is based on very short (1 ns) simulations (see above) initiated from the Aβ_10–35_ NMR structure of Lee et al. [88]. These simulations showed that the CHC, the most structured region in the proposed NMR model, partially unfolds as a result of the E22Q mutation. More recent experiments and simulations in Shea group and others on fragments and on the full-length Aβ peptide, however, indicated that the CHC was not the most structured element of the Aβ peptide [335,377,399,401]. Rather, a bend involving residues E22–K28 emerges as the most structured part of the peptide. The simulations presented by Shea and co-authors lead to a model for Aβ monomer deposition onto fibrils that suggests a mechanism for the enhanced aggregation rates seen in the E22Q mutant. Their REMD simulations show that the E22Q mutation does not alter the structure of the 22–28 bend but rather, this mutation leads to a weakening of the interactions between the CHC and this bend. In the WT peptide, their simulations showed that the CHC did not fold into a unique structure but to an ensemble of three to four structures, all aligned with the bend motif and all populated no more than 20% of the time [399]. The mutation enables the CHC to sample conformational space more readily and adopt a structure commensurate with the fibril. Simply reducing the amount of β-structure in the Aβ peptide (hypothesis a) or a random disordering of the CHC (hypothesis c) will not lead to increased aggregation rates. For enhanced aggregation rates, it is necessary for the peptide to adopt a conformation that bears close similarity to the TS structures for fibril deposition. In their simulations, they observe that the E22Q mutation does precisely that: the E22Q mutation induces changes in the CHC away from random coils and to β-strand-like structures seen in the TS ensemble [399,400].

This structural shift upon mutation translates into a decreased free energy barrier to monomer deposition and, as a consequence, leads to an increased aggregation rate. In agreement with experiments, they find that the entropic contribution dominates the difference in the activation free energy barrier between Aβ_15–28_WT and Aβ_15–28_E22Q [399,400]. The origin of this entropic effect can be due to the conformational entropy of the Aβ_15–28_, indicating that there are more TS structures in the E22Q mutant than in the WT sequence. Alternately, the additional entropy might arise from solvation differences in E22 and Q22 residues due to their different hydrophobicity. Interestingly, experiments show that the deposition of the longer and more cytotoxic variant, Aβ_42_-E22Q, is not significantly faster than that of the Aβ_42_ WT counterpart, suggesting that these two variants may play different roles in familial AD. A compelling feature of their model is its ability to explain why monomer deposition proceeds much faster in Aβ_42_ than in Aβ_40_ [395]. Unlike Aβ_40_, monomers of Aβ_42_ contain ordered β-strand structures in their A30–V36 segment. Hence, for Aβ_40_, there is an additional free energy cost for ordering this segment. It follows that the free energy difference is lower in Aβ_42_ than in Aβ_40_, leading to a higher deposition rate [399].

Li and co-workers studied the effect of Taiwan mutation (D7H) on the structures of Aβ utilizing REMD simulations [401]. They used the OPLS parameters for the protein and implicit model for water. For REMD simulation, the number of replicas is 12 for all systems. The lowest and highest temperatures are 270 and 566 K for Aβ40 systems and 270 and 566 K for Aβ_42_ systems (T = 270.0, 290.2, 311.8, 334.5, 358.4, 383.5, 410.1, 438.0, 467.5, 498.5, 531.2, 566.0 K). Each replica was run for 1000 ns and the data were collected every 10 ps. Their data support the experimental finding that D7H retards fibril growth through the same mechanism for Aβ_40_ and Aβ_42_ [401]. Namely, for both species the reduction of the overall β- structure and at the fibril-prone regions is responsible for the slowing down of association. Although the estimation of relative fibril formation rates based on the bending free energy is approximate, it provides complementary insight into the effect of mutations. The 2-fold reduction in fibril formation rates predicted by this approach is in reasonable accord with the experiments [108,369,402]. Once the multiscale simulations were performed, it was demonstrated that basins obtained in implicit solvent remain stable in explicit solvent. This suggests that their results are also valid for explicit aqueous models. They showed that the Taiwan familial disease mutation has little impact on the collision cross section of alloform Aβ peptides, implying that this parameter is not sensitive enough to probe structural changes. Although the collision cross section of D7H was not experimentally measured, their result is in qualitative agreement with the experiments showing that other mutations like D7N, A21G and E22G have no noticeable effect on the collision cross section of both monomers and oligomers [401].

Derreumaux and co-workers investigated the effect of the Tottori familial disease mutation (D7N) on the monomers and dimers of Aβ_40_ and Aβ_42_ in aqueous solution using MD simulations [403]. They used the OPLS-AA parameters for the proteins and the TIP3P model for water. The monomers were centered in octahedron boxes of 66 Å edges containing 4400 water molecules. The dimers were centered in cubic boxes of 90 Å sides containing 29,000 and 26,500 water molecules for the (WT and D7N) Aβ_40_ dimers and the (WT and D7N) Aβ_42_ dimers, respectively. Each monomer and dimer were studied by running 750 and 800 ns MD simulations, respectively, at 300 K. While an increased β-strand from Aβ_42_-WT monomer (21%) to Aβ_40_-WT monomer (6%) correlates with the observation that Aβ_42_ forms fibrils faster than Aβ_40_, a reduction of β-strand content from Aβ_42_-WT dimer (24%) to Aβ_40_-WT dimer (32%), a constant β-strand content for Aβ_40_ dimer upon D7N mutation and a 50% reduction of β-strand content in Aβ_42_ dimer upon D7N mutation do not contradict the basic understanding of the differences between the two alloforms [403]. Indeed, it is well established that other physicochemical factors such as charge and hydrophobicity contribute to enhanced aggregation rates and what counts in explaining aggregation rate variation is not only the total β-strand content but the nucleation energy barrier that the system has to overcome for breaking any competing β-strand alignments, side-chain contacts or loop conformations and adopting the fibril-prone state. Second, Ono et al. by following the secondary structures of Aβ as a function of time, showed that D7N accelerates the conversion from random coil to β-sheet by 10-fold in Aβ_40_ and 5-fold in Aβ_42_ system [110]. Their monomer simulations revealed a 5% and 14% reduction of coil in Aβ_40_ and Aβ_42_ upon mutation. In contrast, their dimer simulations showed that either the coil content remained constant in Aβ_40_ or increased by 16% in Aβ_42_ upon D7N mutation. Because of CD being averaging structures and since the solution consists of monomers and dimers in equilibrium with larger aggregates, it is possible that dimeric structures exist with a richer coil-composition. Third, Ono et al. proposed that the D7N mutation may affect the Ser8–Gly9 turn and from the effect on this turn, D7N may impact considerably the conformation of the N-terminus and the overall peptide assembly [110]. This is supported by simulations conducted for all D7N species with an increased turn propensity at residues 5–12 (Aβ_40_ monomer) and 1–9 (Aβ_42_ monomer), as well as a decreased turn propensity at residues 6–9 in both dimers [403].

The interplay between the salt bridges Asp1–Arg5 and Asp1–Lys16 is particularly interesting since Lys16 has been shown to play a key role in Aβ assembly, it is a target for Alzheimer’s therapy and a novel Aβ-K16N peptide has been discovered. Charge removal is also accompanied by an increased number of free energy minima for the dimers, with topologies differing from the WT ensemble and a decrease in the β-strand propensity of the N-terminus (residues 7–12) of the Aβ_42_ dimer. Since this N-terminal strand was observed by solid-state NMR spectroscopy for a highly synaptoxic β-amyloid Aβ_40_ oligomer structure, this suggests that the pathogenic conformation varies from WT to D7N [403]. Finally, their data supported the experimental finding that the Tottori mutation accelerates fibril formation of Aβ_40_ and Aβ_42_ peptides [403]. For both systems, they did not find an increase in β-strand content upon mutation. Rather they found that the enhanced formation rate of Aβ_40_ fibrils comes essentially from the formation of the loop Asp23–Lys28 in the monomer, although the propensity of the region 21–30 to adopt turn is enhanced in both the monomer and dimer upon mutation and the population of fibrillar-like states, albeit marginal, is certainly higher in D7N dimer than in WT dimer [403].

Li and co-workers investigated the effect of the English familial disease mutation (H6R) on the structures of monomeric and dimeric Aβ_40_ and Aβ_42_ [404]. They used the OPLS-AA force field parameters for the protein and the TIP4P model for water. In the simulations of Aβ_40/42_ monomers, the species were centered in octahedron boxes of 57 Å with periodic boundary conditions containing about 4400 water molecules. For dimers, they used cubic boxes (the box size is 95 Å for Aβ_40_ dimer and 92 Å for Aβ_42_ dimers) with periodic boundary conditions that contain around 29,000 and 27,000 water molecules for the Aβ_40_ dimers and the Aβ_42_ dimers, respectively. Each monomer was studied by 750 ns MD and each dimer by 800 ns MD at 300 K. Upon mutation, the solvation free energy increases by an amount of about 92 and 194 kcal mol^−1^ for Aβ_40_ and Aβ_42_, respectively. This pronounced effect clearly supported the acceleration of peptide self-assembly by increased hydrophobicity. Their data supported the experimental finding that H6R speeds up fibril growth but the mechanisms are different for Aβ_40_ and Aβ_42_ [110,404]. For both systems, they did not find an increase in overall β-strand content upon mutation. Rather they found that the enhanced aggregation rate of Aβ_42_ comes essentially from the local increase of β-content at the C-terminus for both monomeric and dimeric systems (β-structure of residues 30–42 levels up from 22% to 36% for monomer and from 22% to 51% for dimer) and from the mutation-induced rigidity of the salt bridge between the residues 23 and 28 of monomer. Without mutation, this salt bridge is not formed during MD simulations but in the presence of mutation, its population becomes 90%. In contrast, the enhanced aggregation rate of Aβ_40_ is associated with the increase in turn structure by 30% at residues 26–29 in the turn region and coil reduction at positions 10–13, 26–29 and 30–34 of the monomer as well as with decreased intramolecular salt bridge 23–28 distance in the dimer. They have shown that the English familial disease mutation has little impact on the collision cross section of alloform Aβ peptides, implying that this parameter is not sensitive enough to probe structural changes. Although collision cross section of H6R was not experimentally measured, their result is in qualitative agreement with the experiments, showing that other mutations like D7N, A21G and E22G have no noticeable effect on the collision cross section of both monomers and oligomers [404].

## 4. Aβ and αS Interactions: Insights from the Molecular Dynamics Simulations

Studies suggest that αS may also have a crucial role in the pathology of AD. A large number of AD patients exhibit αS positive Lewy bodies associated with LBD in their brains [405,406]. Evidences show that Aβ and αS interact in vivo and in vitro [407,408,409,410]. Transgenic mouse models demonstrate Aβ enhances αS accumulation and neuronal deficit [408]. Multi-dimensional NMR studies in membrane mimicking environment reported that the molecular interaction of αS with Aβ40 and Aβ42 are site-specific and that membrane bound αS induced structural alterations that are more profound in Aβ42 compared to those in Aβ40 [407]. The same study also suggests that the oligomerization pathways for αS with Aβ42 and Aβ40 in the vicinity of cellular membranes are different [407]. Simulations by Tsigelny et al. [409] showed that Aβ and αS localized on a lipid bilayer surface are capable of forming ring-like hybrid structures that can make a pore in the membrane. Jose and co-workers used atomistic molecular dynamics simulations to probe the possibility of cross dimerization between αS1–95 and Aβ1–42 and thereby gained insights into plausible early assembly pathways in aqueous environment [411]. Their analyses indicate a strong probability of association between the two sequences, with inter-protein attractive electrostatic interactions playing dominant roles. Principal component analysis revealed significant heterogeneity in the strength and nature of the associations in the key interaction modes. In most, the interactions of repeating Lys residues, mainly in the imperfect repeats “KTKEGV” present in αS1–95 were found to be essential for cross interactions and formation of inter-protein salt bridges. Additionally, a hydrophobicity driven interaction mode devoid of salt bridges, where the NAC region of αS1–95 came in contact with the hydrophobic core of Aβ1–42 was observed. The existence of such hetero complexes and therefore hetero assembly pathways may lead to polymorphic aggregates with variations in pathological attributes. Yet, the toxic β-sheet-rich oligomers that initiate the formation of amyloid plaques from Aβ and lead to the formation of LBs from AS were not studied until Raz and Miller conducted MD simulations using the CHARMM22/CMAP parameters and the TIP3P water model [412]. Simulations were run for 60 ns. The cross-seeded NAC Aβ oligomers demonstrated polymorphism. Although NAC-Aβ oligomers exhibit polymorphism, analysis of the populations showed that NAC oligomers prefer to interact with Aβ oligomers to form double- over single-layer conformations. They also found in the cross-seeded NAC-Aβ oligomers, the self-assembled NAC oligomers that consist of three β-strands connected by two turn regions affect the secondary structure of the self-assembled Aβ oligomers. Artificial mutation studies using molecular dynamics simulations remain to be performed on Aβ and αS interactions for understanding their interactions at the atomic level with dynamics.

We should mention here that classical MD simulations can treat up to 10^6^–10^8^ atoms and that the inclusion of the full-size in vivo environment is currently impossible in such simulations. However, MD simulations have also provided useful information for Aβ or αS interactions with other species, such as membranes, amylin, ATP, or transition metal ions (see, for example, [153,154,155,208,320,413,414]).

## 5. Conclusions

We have reviewed what molecular dynamics simulations can tell us about the α-synuclein and amyloid-β proteins and how artificial and pathological mutations impact the structures of α-synuclein and amyloid-β proteins linked to Parkinson’s and Alzheimer’s diseases. Our knowledge of the structures of αS and Aβ_40/42_ fibrils, protofibrils and large oligomers has markedly increased in recent years and it is clear that polymorphism is present from the monomer to fibrils. We know that fibrils with different molecular structures can result from environment-dependent self-assembly and kinetic rather than thermodynamic control. We also know that metastable states can be alleviated by using appropriate seeds or under shear flow and the structural models of fibrils that build up take on different structures in the brain of diseased Parkinson’s or Alzheimer’s patients with different PD/AD symptoms. This high degree of polymorphism, which arises from many physical factors and persists in vitro and in brain tissues, is correlated to different phenotypes and is rather bad news for drug design because one drug may be efficient for one patient but not for another.

Structural and dynamical characterization of the smallest oligomers, the most toxic species and the monomers has been moving at a slower pace due to their transient character and intrinsic disorders but with the help of new experimental methods and efficient sampling methods using multiple force fields and representations, our knowledge of these species in aqueous solution, in proximity to or in the membranes should significantly increase, although polymorphism of the aggregates and high sensitivity to external conditions humpers the reproducibility of the experimental readouts and the convergence of the simulations. One particular advantage of computer simulations, however, is that calculations can be repeated using different pH condition and model membranes and the effects of site-specific artificial or pathological mutations can be investigated.

Characterizations of the primary nucleus/nuclei and the population of the monomeric state prior to the lag phase remain difficult both experimentally and theoretically due to the sensitivity to the experimental conditions and the amino acid sequence. One amino acid substitution is sufficient to change the free energy landscape as evidenced from the kinetics and the oligomer size distribution of FPD and FAD Molecular dynamics simulations provide insights onto the changes of the structures and free energy landscapes (thermodynamic properties) at the atomic level with dynamics. How the structures of αS and Aβ may relate to the mechanism of toxicity is still unknown since toxicity comes from all monomers, oligomers to the fibrils. One source of toxicity comes from membrane channel formation and the cylindroid conformation has been suggested to be toxic but other antiparallel β-sheet conformations are also considered toxic. In addition, a single amino acid change is able either to reduce or to increase toxicity.

Despite extensive studies, drug after drug aimed at targeting αS and Aβ has failed to slow the progression of PD and/or AD in clinical trials. If it is true that we are treating people too late, there are however two other hurdles for drug improvement. First, while many groups are working on developing drugs that bind to αS and Aβ fibrils (therefore reducing the fragmentation process) or bind to αS and Aβ oligomers to slow or accelerate fibrillation and in all cases reduce αS and Aβ cytotoxicity, how they interact with αS and Aβ remains to be unknown at an atomic resolution, yet obtaining high-resolution structures of the αS/Aβ oligomer/drug complexes is a prerequisite to optimizing the kinetic and thermodynamic binding properties of promising compounds (and thus their specificity), prior to cell viability essays, animal models for PD/AD and clinical trials. The second hurdle is that repeated identification of the same types of molecules as promising hits against different proteins is polluting the chemical literature.

While many inhibitors have been designed to target a specific region of αS/Aβ, it would be interesting to study in cells the cumulative effect of inhibitors designed to recognize different regions of αS/Aβ. It would also be of great interest to combine different drugs targeting αS/Aβ processing and rendering αS/Aβ aggregates very unstable and more prone to degradation. Today, we are just seeing the tip of the iceberg in understanding phenotype-related toxicity and aggregation propensity of WT αS/Aβ and its familial disease and protective variants but continuous and synergetic efforts between in vitro and in vivo studies (including basic verifications such as purity and reproducibility of the results using various readouts or transgenic animals with different sexes and times of PD/AD incubation) and theoretical studies (using multiple approaches) should get us closer to finding a cure for PD/AD. Pathological mutations change the structures and thermodynamic properties of αS/Aβ, indicating that different drugs need to be considered for treating PD/AD.

## Figures and Tables

**Table 1:** Calculated average enthalpy (*H*), entropy (*TS*) and Gibbs free energy (*G*) values for the WT, A53T mutant, A30 P mutant and E46 K mutant type αS proteins in an aqueous solution medium.

Protein	<*H*> (kJ·mol^−1^)	−*T*<*S*_NMA_> (kJ·mol^−1^)	<*G*_MM/PBSA_> (kJ·mol^−1^)
WT αS	−9557.4 (±18.4)	−7043.8 (±8.4)	−16,601.4 (±13.0)
A53T αS	−9662.3 (±21.0)	−7072.0 (±9.6)	−16,734.3 (±10.2)
A30P αS	−9461.4 (±50.8)	−7057.3 (±18.8)	−16,518.7 (±32.1)
E46K αS	−9495.6 (±60.9)	−6991.3 (±13.7)	−16,486.9 (±54.2)

**Table 2:** The calculated average enthalpy (*H*), entropy (*S*) and Gibbs free energy (*G*) values for the WT and R5A mutant Aβ_42_ peptides in aqueous solution using harmonic and quasi-harmonic methods.

Protein	<*H*> (kJ·mol^−1^)	−*T*<*S*_NMA_> (kJ·mol^−1^)	−*T*<S*_QH_*> (kJ·mol^−1^)	<*G*_NMA_>	<*G*_QH_>
WT Aβ42	−2612.9 (±127.4)	−2196.4 (±39.3)	−5895.3 (±18.0)	−4809.3 (±126.3)	−8508.2 (±128.7)
R5A Aβ42	−1736.9 (±127.9)	−2196.8 (±43.5)	−6151.3 (±44.3)	−3933.6 (±125.0)	−7888.2 (±135.4)

**Table 3:** Salt bridges formed in WT Aβ_42_ and the Tyr10Ala mutant in aqueous solution.

DONOR	Acceptor	WT Aβ_42_ (P %)			Tyr10Ala Aβ_42_ (P %)		
		R(C-N) ≤ 4 Å	R(C-N) ≤ 5 Å	R(C-N) ≤ 6 Å	R(C-N) ≤ 4 Å	R(C-N) ≤ 5 Å	R(C-N) ≤ 6 Å
R5	E3	57.7	59.2	59.6	61.2	64.3	65.5
R5	E22	26.4	26.7	26.7	9.3	9.8	10.0
R5	A42 (-COO^−^)	20.2	21.0	21.3	20.0	20.7	21.2
R5	E11	14.1	15.9	16.7	16.6	19.3	20.4
R5	D1	13.9	14.8	15.0	18.3	19.5	20.9
R5	D23	8.2	8.4	8.4	10.9	12.7	13.4
K28	E22	7.3	11.6	13.2	3.9	6.6	7.4
K16	A42 (-COO^−^)	5.6	8.1	9.4	4.2	5.7	6.3
K28	D23	3.9	6.2	7.1	7.9	11.4	13.2
K16	E11	3.8	6.7	8.0	10.9	19.1	22.8
K16	D7	3.8	8.3	11.1	7.0	9.3	9.9
D1 (-NH_3_^+^)	E3	2.8	4.5	5.3	2.0	2.8	3.1
K16	D23	1.9	3.6	4.1	0.3	0.6	0.7
K28	A42 (-COO^−^)	1.8	2.6	2.9	1.8	2.6	2.8
K16	E3	0.9	1.6	1.8	1.6	2.5	2.9
R5	D7	0.7	0.9	1.2	4.2	5.0	5.9
K16	D1	0.7	0.9	1.0	1.8	2.9	3.1
K28	D1	–	–	–	4.2	5.9	6.3

**Table 4:** Calculated mean values of enthalpy (*H*), entropy (*TS*) and Gibbs free energy (*G*) for the WT and ΔE22 mutant Aβ_40_ and Aβ_42_ peptides using the normal mode analysis (*S*_NMA_) and Schlitter (*S*_QM_) methods to calculate the entropic contribution to *G*.

Peptide	<*H*> (kJ·mol^−1^)	−*T*<*S*_NMA_> (kJ·mol^−1^)	<*S*_QH_> (kJ·mol^−1^)	<*G*_NMA_> (kJ·mol^−1^)	<*G*_QH_> (kJ·mol^−1^)
WT Aβ40	−2788.2 (±55.6)	−2114.4 (±9.9)	−5334.3 (±78.2)	−4902.5 (±45.9)	−8361.5 (±95.9)
WT Aβ42	−2579.9 (±24.2)	−2206.6 (±4.1)	−5781.3 (±131.3)	−4786.5 (±20.3)	−8122.2 (±93.5)
ΔE22 Aβ40	−2202.7 (±41.0)	−2177.7 (±10.5)	−5810.1 (±123.3)	−4380.4 (±30.7)	−7821.8 (±99.9)
ΔE22 Aβ42	−2454.9 (±38.3)	−2072.9 (±8.7)	−5367.0 (±86.6)	−4526.6 (±30.5)	−8012.7 (±94.7)
